# Recreational and sexualised drug use among gay, bisexual, and other men who have sex with men (gbMSM) in Ireland–Findings from the European MSM internet survey (EMIS) 2017

**DOI:** 10.1371/journal.pone.0288171

**Published:** 2023-07-28

**Authors:** Fionn P. Daly, Kate O’Donnell, Martin P. Davoren, Chris Noone, Peter Weatherburn, Mick Quinlan, Bill Foley, Fiona Lyons, Derval Igoe, Peter Barrett

**Affiliations:** 1 School of Medicine and Health, University College Cork, Cork, Ireland; 2 School of Public Health, University College Cork, Cork, Ireland; 3 HSE Health Protection Surveillance Centre, Dublin, Ireland; 4 Sexual Health Centre, Cork, Ireland; 5 School of Psychology, National University of Ireland Galway, Galway, Ireland; 6 Sigma Research, London School of Hygiene and Tropical Medicine, London, United Kingdom; 7 Gay Health Network, Dublin, Ireland; 8 St. James’s Hospital, James St, Saint James’ (part of Phoenix Park), Dublin 8, Dublin, Ireland; 9 Department of Public Health HSE-South, St. Finbarr’s Hospital, Cork, Ireland; University of Technology Sydney, AUSTRALIA

## Abstract

**Background:**

Gay, bisexual, and other men who have sex with men (gbMSM) report a higher prevalence of drug use in comparison to the general male population. However, in Ireland, there is a paucity of literature regarding the prevalence of drug use and its determinants among gbMSM.

**Aims/Objectives:**

To quantify the prevalence of (i) recreational drug use (RDU) and (ii) sexualised drug use (SDU) among gbMSM in Ireland, and to identify the factors associated with these drug use practices.

**Methods:**

The European MSM Internet Survey (EMIS) 2017 was an online, anonymous, internationally-promoted questionnaire. Two binary outcomes were included in our analyses: (1) RDU and (2) SDU in the previous year. Multivariable-adjusted logistic regression explored factors associated with these outcomes, and all independent covariates were adjusted for one another.

**Results:**

Among gbMSM without HIV (n = 1,898), 40.9% and 13.1% engaged in RDU and SDU in the previous year, respectively. Among diagnosed-positive gbMSM (n = 141), the past-year respective prevalence estimates were 51.8% and 26.2%. Increased odds of RDU were observed among gbMSM who were younger (vs. 40+ years) (18–24 years; AOR 2.96, 95% CI 2.05–4.28, 25–39 years; AOR 1.66, 95% CI 1.27–2.16), lived in Dublin (vs. elsewhere) (AOR 1.47, 95% CI 1.17–1.83), and engaged in condomless anal intercourse (CAI) in the previous year (vs. none) (1–2 partners; AOR 1.79, 95% CI 1.34–2.38, 6+ partners; AOR 1.79, 95% CI 1.18–2.71). Greater odds of SDU were identified among those who lived in Dublin (vs. elsewhere) (AOR 1.50, 95% CI 1.07–2.10), and engaged in CAI (vs. none) (1–2 partners; AOR 3.16, 95% CI 2.05–4.88, 3–5 partners; AOR 2.50, 95% CI 1.47–4.26, and 6+ partners; AOR 3.79, 95% CI 2.23–6.43).

**Conclusion:**

GbMSM report a high prevalence of drug use in Ireland. Targeted interventions, including harm reduction campaigns, may be needed to support healthier drug use choices among this community.

## 1. Introduction

In 2020, approximately 284 million people worldwide reported the use of illicit drugs within the previous 12 months; a 26% increase from 2010 [1]. Drug-related harms can have deleterious effects on one’s life [2]. For example, drug use is often associated with an increased risk of fatal poisonings and seizures, behavioural harms (such as accidents, violence, and crime), the acquisition of sexually transmitted infections (STIs) and human immunodeficiency virus (HIV), and a multitude of adverse physical and mental health outcomes [3–5].

In Ireland, the overall prevalence of recreational drug use (RDU) has risen from 5.6% in 2002/2003 to 7.4% in 2019/2020 [6, 7], and in 2019/2020, males reported a higher prevalence in comparison to females (12.3% and 5.7%, respectively) [7]. International evidence suggests that gay, bisexual, and other men who have sex with men (gbMSM) have a higher prevalence of drug use versus age-comparable non-gbMSM [8–10]. In general, gbMSM already face considerable health-related inequalities in society, particularly those which pertain to sexual health and mental health [11]. As drug use can overlap with these domains [12], it may therefore be detrimental to the overall wellbeing of gbMSM, and could result in a range of associated harms [13, 14]. Previously cited reasons for this drug use disparity may include “minority stress” [14], as well as the perceived “normalisation” of drug use within the gbMSM community [15], including sexualised drug use (SDU) [16].

“Chemsex”, a subset of SDU [17], is a term which is specific to the gbMSM community, and is ascribed to the utilisation of certain drugs to facilitate and enhance sexual encounters [18], which often occur in group sessions [19]. Drugs commonly included in the definition of chemsex are gamma-hydroxybutyrate/gamma-butyrolactone (GHB/GBL), mephedrone, crystal methamphetamine, and to a lesser extent, ketamine [20–22]. A body of evidence proposes that chemsex may be associated with an array of adverse mental, physical, and/or sexual health outcomes [19, 23–27]. For example, all four of these drugs can also induce intense feelings of sexual arousal [25], which may lead to extreme sexual disinhibition and enhance longevity of sexual contact [28]. Safer sex practices can be less important to those under the influence of these drugs which may, consequently, lead to increased acquisition of STIs and onward transmission of HIV [29]. In relation to mental health outcomes, mephedrone and crystal methamphetamine frequently cause extreme anxiety and paranoia in the acute setting, which often precede prolonged bouts of depression [23–25].

Currently, there is a scarcity of literature examining both RDU and SDU among gbMSM in Ireland. The most recently estimated national prevalence of RDU among gbMSM in Ireland is 36% [30]. However, these data are from 2015, and more recent estimates are warranted, hence the need for our study. Additionally, previous estimates of SDU among gbMSM in Ireland have ranged from as low as 6% [21] and 7% [30] in national surveys, to 27% among gbMSM-specific sexual health clinic attendees [31]. Our study aimed to ascertain prevalence estimates using the latest available national Irish data, and we sought to identify the various demographic, psychosocial, and behavioural characteristics of gbMSM who engage in RDU and SDU. The purpose of this is to identify certain subgroups who are likely to benefit from targeted messaging, which may curtail the potential harms that can be associated with these drug use practices.

## 2. Methods

### 2.1. Study design

We utilised the Irish dataset from the European MSM Internet Survey (EMIS) 2017. EMIS-2017 was an online, anonymous, internationally-promoted survey among gbMSM across 50 countries in Europe, and was adapted from a previous survey, EMIS-2010 [32]. Novel questions were added to EMIS-2017, and were based on topical developments in certain areas which affect the overall health and wellbeing of gbMSM. For example, questions relating to the availability and use of pre-exposure prophylaxis (PrEP) were included in EMIS-2017 [33]. The survey, which was available in any one of 33 different languages, began on 18^th^ October 2017 and ended on 31^st^ January 2018. Overall, 409 items were included in the survey, and a broad range of areas were explored, including sociodemographic characteristics, mental health, sexual health, and substance use [34].

### 2.2. Study population and sampling

In order to meet inclusion criteria for this study, participants had to initially give informed (written) consent. Respondents were required to identify as either a man and/or a trans man, and had to be sexually attracted to, or sexually active with, men. For inclusion in the Irish dataset specifically, candidates had to be resident in the Republic of Ireland, regardless of their native country (gbMSM residing in Northern Ireland were excluded). In addition, the study population were required to be over the legal age of sexual consent in their respective countries, i.e. at least 17 years of age in Ireland. However, we restricted our study sample to those aged 18+ years in accordance with the Social Research Ethics Committee (SREC) policies at University College Cork (UCC).

In order to recruit participants, a multi-media strategy was utilised [35], including:

1. Statutory and non-governmental organisation (NGO) websites for gbMSM.
2. Geospatial networking/contact applications, including Scruff, Gaydar, and GROWLr.
3. Multiple social media platforms, such as Instagram, Twitter, and Facebook.

There was also widespread promotion of EMIS-2017 through lesbian, gay, bisexual, and transgender+ (LGBT+) community organisations, along with the issuing of a press release for print media. Finally, business cards and posters were distributed at gay social/community venues.

### 2.3. Measurements

#### 2.3.1. Outcomes

Our two primary outcomes of interest were:

1. Any RDU (excluding poppers) in the previous 12 months (yes/no).
2. Engagement in SDU in the previous 12 months (yes/no).

EMIS-2017 contained a series of questions which related to drug use, including the specific type(s) of drug(s) the participants had ever used, and the recency of drug use (if ever). From this, we derived the first binary outcome variable. The full list of drugs which were used to derive this variable may be found below (results; section 3). Poppers were excluded as prior surveys have elucidated a higher prevalence of their use among gbMSM in comparison to other drugs, and because the legality pertaining to their use is not entirely clear in Ireland [30, 36].

A separate question asked participants whether they had ever used a pre-specified list of drugs (specifically, MDMA/“ecstasy”, cocaine, amphetamine/“speed”, crystal methamphetamine, mephedrone, and/or ketamine) *“to make sex more intense or last longer”* (i.e. SDU). If so, then respondents were asked about the recency of their engagement in SDU. From this, we derived the second binary outcome variable. Importantly, EMIS-2017 did not include GHB/GBL in this SDU-related question, unlike a previous Irish survey of gbMSM [37].

#### 2.3.2. Independent covariates

All included covariates were identified *a priori*. The selection of covariates was informed by a scoping review of the literature. A preliminary list was circulated amongst a panel of national stakeholders, and the final selection was achieved through consensus-decision making.

Although age was collected as a continuous variable, it was analysed categorically, and recoded it into three categories: 40+ years, 25–39 years, or 18–24 years. The rationale for this decision was that SDU (specifically chemsex) tends to be most common among gbMSM in their late twenties, thirties, and early forties [21, 37]. Therefore, expressing this covariate as a continuous one may mask a potential “n-shaped” association [38]. This contrasts with RDU, which tends to peak in the earlier years, and is typically most common in the 18–24 age group [37, 39].

Number of condomless anal intercourse (CAI) partners in the previous year referred only to non-steady male partners. Although it was collected as a continuous variable, it was analysed categorically, and was recoded into four categories: 0, 1–2, 3–5, or 6+ partners. Furthermore, a proxy was created for “group sex”, as men were asked about the details of their last sexual encounter. Specifically, respondents were asked the following question: “were you having sex with one man or more than one man?”. From this, a new variable was generated, and assessed whether participants’ last sexual encounter was with 2+ males. Finally, EMIS-2017 utilised the “patient health questionnaire-4” (PHQ-4), a validated four-item inventory which is rated on a four-point Likert-type scale, to screen for anxiety/depression [40]. This screening tool draws the first two items from the “generalised anxiety disorder-7 scale” (GAD-7) [41], as well as the “patient health questionnaire-8” (PHQ-8) [42], to screen for adverse mental health among the study sample. Details on the coding and analysis of the remaining covariates can be found in the supporting information (S1 Appendix).

### 2.4. Statistical analysis

Descriptive statistics were used to compare baseline characteristics of the study sample. Absolute numbers and percentages were reported for categorical variables. Pearson’s chi-square (χ^2^) was utilised for non-parametric tests of bivariate associations. Fisher’s exact test was employed for categorical covariates with a count of <5 in >20% of cells (i.e. Table 2) [43].

Single-predictor logistic regression analysis was initially conducted to calculate crude measures of association between independent variables and dichotomous outcomes. Subsequently, multivariable-adjusted logistic regression analysis was used in order to identify which independent variables were robust to adjustment with an array of potentially confounding covariates. All independent covariates included in these models were adjusted for one another. As we were dealing with cross-sectional data, our intention was to explore associations, without determining any causal relationships. Multicollinearity between explanatory variables was assessed using Spearman’s rank correlation coefficient, wherein all values were below the often-cited threshold of 0.40, indicating that multicollinearity was unlikely to be an issue [44]. Furthermore, when missing data for a particular categorical variable are small (i.e. <5%), biases and loss of statistical power are likely to be relatively inconsequential [45]. Thus, where 5% or more data were missing for an independent categorical variable, indicator variables were synthesised and included in the regression models.

Our analysis was stratified based on HIV status, with a separate sensitivity analysis of HIV-positive respondents using descriptive statistics and regression analyses for RDU and SDU outcomes. Variables pertaining to HIV testing history and current pre-exposure prophylaxis (PrEP) use were not included in these models, as all men were diagnosed HIV-positive, which obviated the requirement for their inclusion. This approach was determined *a priori* in conjunction with multi-agency groups who work to promote the overall health and wellbeing of gbMSM.

All analysis was undertaken using Stata version 17.0 (StataCorp., TX, USA). Two-sided significance tests were utilised throughout, and all p-values were tested against a pre-set alpha level of significance which was set at 5%.

### 2.5. Ethical considerations

The Observational Research Ethics Committee at the London School of Hygiene and Tropical Medicine (LSHTM) originally granted ethical approval for EMIS-2017. For this study specifically, the Social Research Ethics Committee (SREC) in University College Cork (UCC) granted approval, wherein LSHTM and UCC both approved a Data Transfer Agreement (S2 Appendix).

## 3. Results

Tables 1 and 2 present the baseline characteristics of non-HIV diagnosed and HIV-diagnosed gbMSM, respectively.

**Table 1:** Baseline characteristics of gbMSM without a diagnosis of HIV who took part in EMIS-2017 and were included in the study sample, by engagement in recreational drug use (excluding poppers) and sexualised drug use in the previous 12 months (n = 1,898).

	RDU (%)	None (%)	P-value	SDU (%)	None (%)	P-value
**Age → n (%)**			**<0.001**			**0.01**
40+	161 (20.8)	405 (36.1)		61 (24.5)	505 (30.6)	
25–39	389 (50.1)	500 (44.6)		138 (55.4)	751 (45.6)	
18–24	226 (29.1)	217 (19.3)		50 (20.1)	393 (23.8)	
**Education >16 years → n (%)**			0.19			0.31
0–3 years	148 (19.1)	232 (20.7)		47 (18.9)	333 (20.2)	
4–6 years	320 (41.2)	409 (36.4)		106 (42.6)	623 (37.8)	
+7 years	237 (30.5)	378 (33.7)		70 (28.1)	545 (33.0)	
*Missing ^*	*71 (9*.*2)*	*103 (9*.*2)*		*26 (10*.*4)*	*148 (9*.*0)*	
**Employment → n (%)**			**<0.001**			**0.01**
Employed	551 (71.3)	828 (74.0)		202 (81.8)	1177 (71.6)	
Unemployed	36 (4.6)	48 (4.3)		10 (4.1)	74 (4.5)	
Student	167 (21.6)	179 (16.0)		30 (12.1)	316 (19.2)	
Other **+**	19 (2.5)	64 (5.7)		5 (2.0)	78 (4.7)	
**Area of residence → n (%)**			**<0.001**			**0.001**
Outside Dublin	271 (34.9)	506 (45.1)		76 (30.5)	701 (42.5)	
Dublin	462 (59.6)	549 (48.9)		159 (63.9)	852 (51.6)	
*Missing ^*	*43 (5*.*5)*	*67 (6*.*0)*		*14 (5*.*6)*	*96 (5*.*9)*	
**Born in Ireland → n (%)**			0.74			0.63
No	193 (25.0)	272 (24.3)		64 (25.8)	401 (24.4)	
Yes	580 (75.0)	848 (75.7)		184 (74.2)	1244 (75.6)	
**Sexual identity → n (%)**			0.36			0.33
Gay/Homosexual	633 (81.6)	901 (80.3)		208 (83.5)	1326 (80.4)	
Bisexual	104 (13.4)	147 (13.1)		31 (12.5)	220 (13.3)	
Other †	39 (5.0)	74 (6.6)		10 (4.0)	103 (6.3)	
**Outness → n (%)**			**<0.001**			0.14
None	29 (3.8)	99 (9.0)		10 (4.0)	118 (7.3)	
Less than half	165 (21.5)	322 (29.3)		61 (24.6)	426 (26.3)	
More than half	143 (18.6)	186 (16.9)		41 (16.5)	288 (17.8)	
All or almost all	431 (56.1)	493 (44.8)		136 (54.9)	788 (48.6)	
**Currently using PrEP → n (%)**			**0.001**			**<0.001**
No	724 (94.1)	1086 (97.2)		220 (89.8)	1590 (96.9)	
Yes	45 (5.9)	31 (2.8)		25 (10.2)	51 (3.1)	
**HIV testing history → n (%)**			**0.001**			**<0.001**
Never tested	152 (19.7)	296 (26.6)		29 (11.7)	419 (25.6)	
Last HIV test negative	620 (80.3)	818 (73.4)		219 (88.3)	1219 (74.4)	
**CAI in last 12 months → n (%)**			**<0.001**			**<0.001**
0	388 (52.4)	744 (68.7)		83 (34.9)	1049 (66.1)	
1–2	187 (25.2)	182 (16.8)		71 (29.8)	298 (18.8)	
3–5	77 (10.4)	88 (8.1)		33 (13.9)	132 (8.3)	
6+	89 (12.0)	69 (6.4)		51 (21.4)	107 (6.8)	
**Last sex encounter was group sex → n (%)**			**<0.001**			**<0.001**
No	506 (65.2)	630 (56.1)		152 (61.0)	984 (59.7)	
Yes	103 (13.3)	131 (11.7)		54 (21.7)	180 (10.9)	
*Missing* ^	*167 (21*.*5)*	*361 (32*.*2)*		*43 (17*.*3)*	*485 (29*.*4)*	
**STI in last 12 months → n (%)**			**<0.001**			**<0.001**
No	603 (80.6)	987 (90.2)		164 (68.3)	1426 (89.0)	
Yes	145 (19.4)	107 (9.8)		76 (31.7)	176 (11.0)	
**Anxiety/Depression → n (%)**			**<0.001**			0.09
None	284 (37.0)	526 (47.5)		98 (40.0)	712 (43.7)	
Mild	300 (39.1)	359 (32.5)		86 (35.1)	573 (35.2)	
Moderate	103 (13.4)	129 (11.7)		42 (17.1)	190 (11.7)	
Severe	81 (10.5)	92 (8.3)		19 (7.8)	154 (9.4)	

^: Indicator variables were synthesised for categorical variables with ≥5% missing data.

+: Retired, on long-term sick leave, or other.

†: All other terms, or does not use a term.

GbMSM = Gay, bisexual, and other men who have sex with men; HIV = Human immunodeficiency virus; EMIS = European MSM Internet Survey; AOR = Adjusted odds ratio; 95% CI = 95% confidence interval; RDU = Recreational drug use; PrEP = Pre-exposure prophylaxis; CAI = Condomless anal intercourse; STI = Sexually transmitted infection.

**Table 2:** Baseline characteristics of gbMSM with a diagnosis of HIV who took part in EMIS-2017 and were included in the study sample, by engagement in recreational drug use (excluding poppers) and sexualised drug use in the previous 12 months (n = 141).

	RDU (%)	None (%)	P-value	SDU (%)	None (%)	P-value
**Age → n (%)**			0.42			0.94
40+	33 (45.2)	38 (55.9)		18 (48.7)	53 (51.0)	
25–39	38 (52.1)	29 (42.6)		18 (48.7)	49 (47.1)	
18–24	2 (2.7)	1 (1.5)		1 (2.7)	2 (1.9)	
**Education >16 years → n (%)**			0.06			0.52
0–3 years	11 (15.1)	17 (25.0)		6 (16.2)	22 (21.1)	
4–6 years	23 (31.5)	28 (41.2)		11 (29.7)	40 (38.5)	
+7 years	34 (46.6)	17 (25.0)		17 (46.0)	34 (32.7)	
*Missing* ^	*5 (6*.*8)*	*6 (8*.*8)*		*3 (8*.*1)*	*8 (7*.*7)*	
**Employment → n (%)**			0.71			0.18
Employed	58 (81.7)	52 (76.5)		32 (86.5)	78 (76.5)	
Unemployed	4 (5.6)	4 (5.9)		3 (8.1)	5 (4.9)	
Student	3 (4.2)	2 (2.9)		1 (2.7)	4 (3.9)	
Other +	6 (8.5)	10 (14.7)		1 (2.7)	15 (14.7)	
**Area of residence → n (%)**			**0.03**			**<0.001**
Outside Dublin	19 (26.0)	31 (45.6)		6 (16.2)	44 (42.3)	
Dublin	48 (65.8)	35 (51.5)		25 (67.6)	58 (55.8)	
*Missing* ^	*6 (8*.*2)*	*2 (2*.*9)*		*6 (16*.*2)*	*2 (1*.*9)*	
**Born in Ireland → n (%)**			0.24			0.15
No	26 (35.6)	18 (26.5)		15 (40.5)	29 (27.9)	
Yes	47 (64.4)	50 (73.5)		22 (59.5)	75 (72.1)	
**Sexual identity → n (%)**			**0.02**			0.19
Gay/Homosexual	69 (94.5)	56 (82.4)		36 (97.3)	89 (85.6)	
Bisexual	0 (0.0)	6 (8.8)		0 (0.0)	6 (5.8)	
Other †	4 (5.5)	6 (8.8)		1 (2.7)	9 (8.6)	
**Outness → n (%)**			**0.02**			0.33
None	0 (0.0)	1 (1.5)		0 (0.0)	1 (1.0)	
Less than half	8 (11.0)	20 (29.9)		4 (10.8)	24 (23.3)	
More than half	11 (15.0)	7 (10.4)		6 (16.2)	12 (11.6)	
All or almost all	54 (74.0)	39 (58.2)		27 (73.0)	66 (64.1)	
**CAI in last 12 months → n (%)**			**0.02**			**<0.001**
0	15 (23.1)	26 (40.0)		2 (5.7)	39 (41.1)	
1–2	10 (15.4)	15 (23.1)		4 (11.4)	21 (22.1)	
3–5	9 (13.8)	10 (15.4)		7 (20.0)	12 (12.6)	
6+	31 (47.7)	14 (21.5)		22 (62.9)	23 (24.2)	
**Last sex encounter was group sex → n (%)**			0.05			**0.004**
No	37 (50.7)	38 (55.9)		18 (48.6)	57 (54.8)	
Yes	26 (35.6)	13 (19.1)		17 (46.0)	22 (21.2)	
*Missing ^*	*10 (13*.*7)*	*17 (25*.*0)*		*2 (5*.*4)*	*25 (24*.*0)*	
**STI in last 12 months → n (%)**			0.27			0.20
No	49 (68.1)	49 (76.6)		23 (63.9)	75 (75.0)	
Yes	23 (31.9)	15 (23.4)		13 (36.1)	25 (25.0)	
**Anxiety/Depression → n (%)**			0.75			0.89
None	28 (38.4)	28 (42.4)		15 (40.6)	41 (40.2)	
Mild	26 (35.6)	23 (34.9)		13 (35.1)	36 (35.3)	
Moderate	7 (9.6)	8 (12.1)		5 (13.5)	10 (9.8)	
Severe	12 (16.4)	7 (10.6)		4 (10.8)	15 (14.7)	

^: Indicator variables were synthesised for categorical variables with ≥5% missing data.

+: Retired, on long-term sick leave, or other.

†: All other terms, or does not use a term.

GbMSM = Gay, bisexual, and other men who have sex with men; HIV = Human immunodeficiency virus; EMIS = European MSM Internet Survey; AOR = Adjusted odds ratio; 95% CI = 95% confidence interval; RDU = Recreational drug use; CAI = Condomless anal intercourse; STI = Sexually transmitted infection.

In total, 1,898 gbMSM (91.1% of the overall study population) were aged 18+ years, had complete drug use data recorded, and had not previously been diagnosed with HIV. The median age was 32 years (range 18–74 years), and 1882 (99.2%) and 16 (0.8%) participants identified as man and trans man, respectively. Overall, 776 (40.9%) and 249 (13.1%) men engaged in RDU and SDU in the previous 12 months, respectively.

Furthermore, 141 (6.8% of the overall study population) were aged 18+ years, had complete drug use data recorded, and had a previous diagnosis of HIV. One hundred and thirty six (95.7%) had an undetectable viral load due to treatment with anti-retroviral therapy (ART). The median age in this group was 40 years (range 21–68 years), 140 (99.3%) and <5 (<3.5%) respondents identified as man and trans man, respectively. In total, 73 (51.8%) and 37 (26.2%) gbMSM with a diagnosis of HIV reported RDU and SDU in the past year, respectively.

The full list of drugs used to derive the RDU variable is as follows: cannabis, cocaine, MDMA pill form, MDMA powder or crystal form, ketamine, amphetamine/speed, GHB/GBL, lysergic acid diethylamide (LSD), crystal methamphetamine, synthetic cannabinoids, mephedrone, synthetic stimulants other than mephedrone (e.g. 3-methylmethcathinone), crack cocaine, and heroin or other related drugs (e.g. fentanyl). The prevalence of past-year use of each specific drug among the study sample is fully outlined in the supporting information (S3 Appendix).

### 3.1. Multi-predictor logistic regression analysis

#### 3.1.1. RDU among gbMSM without a diagnosis of HIV

In our initial multivariable model (Table 3), the odds of engaging in RDU were higher in younger age groups (vs. aged 40+ years) (18–24 years; AOR 2.96, 95% CI 2.05–4.28, 25–39 years; AOR 1.66, 95% CI 1.27–2.16), and in gbMSM who self-identified as bisexual (vs. gay/homosexual) (AOR 1.74, 95% CI 1.22–2.48). Increased odds of RDU were observed among men who disclosed their sexual orientation to a larger number of family/close friends (vs. none) (less than half; AOR 1.72, 95% CI 1.02–2.91, more than half; AOR 2.29, 95% CI 1.31–4.00, all or almost all; AOR 3.20, 95% CI 1.88–5.46), and among those who engaged in CAI with non-steady male partners in the previous 12 months (vs. none) (1–2 partners; AOR 1.79, 95% CI 1.34–2.38, 6+ partners; AOR 1.79, 95% CI 1.18–2.71). Any RDU in the previous year was significantly more likely among gbMSM who self-reported a bacterial STI diagnosis within the same timeframe (vs. none) (AOR 1.53, 95% CI 1.12–2.10), lived in Dublin (vs. outside Dublin) (AOR 1.47, 95% CI 1.17–1.83), and screened positive for a mild degree of anxiety/depression (vs. none) (AOR 1.45, 95% CI 1.14–1.84).

**Table 3:** Factors associated with engagement in recreational drug use (excluding poppers) in the previous 12 months among gbMSM without a diagnosis of HIV who took part in EMIS-2017 and were included in the study sample (n = 1,898)*.

	RDU n (%)	OR	Univariable analysis 95% CI	P-Value	AOR	Multivariable analysis 95% CI	P-Value
**Age**							
40+	161 (28.4)	1	Ref	–	1	Ref	–
25–39	389 (43.8)	**1.96**	**(1.56, 2.45)**	**<0.001**	**1.66**	**(1.27, 2.16)**	**<0.001**
18–24	226 (51.0)	**2.62**	**(2.02, 3.40)**	**<0.001**	**2.96**	**(2.05, 4.28)**	**<0.001**
**Education >16 years**							
0–3 years	148 (38.9)	1	Ref	–	1	Ref	–
4–6 years	320 (43.9)	1.23	(0.95, 1.58)	0.11	1.08	(0.80, 1.44)	0.62
+7 years	237 (38.5)	0.98	(0.76, 1.28)	0.90	0.93	(0.68, 1.27)	0.65
*Missing* ^	*71 (40*.*8)*	*1*.*08*	*(0*.*75*, *1*.*56)*	*0*.*68*	*1*.*11*	*(0*.*73*, *1*.*70)*	*0*.*62*
**Employment**							
Employed	551 (40.0)	1	Ref	–	1	Ref	–
Unemployed	36 (42.9)	1.13	(0.72, 1.76)	0.60	1.26	(0.75, 2.11)	0.38
Student	167 (48.3)	**1.40**	**(1.11, 1.78)**	**0.01**	0.92	(0.66, 1.28)	0.61
Other +	19 (22.9)	**0.45**	**(0.26, 0.75)**	**0.003**	0.58	(0.31, 1.08)	0.09
**Area of residence**							
Outside Dublin	271 (34.9)	1	Ref	–	1	Ref	–
Dublin	462 (45.7)	**1.57**	**(1.30, 1.90)**	**<0.001**	**1.47**	**(1.17, 1.83)**	**0.001**
*Missing ^*	*43 (39*.*1)*	*1*.*20*	*(0*.*79*, *1*.*81)*	*0*.*39*	*1*.*47*	*(0*.*90*, *2*.*41)*	*0*.*13*
**Born in Ireland**							
No	193 (41.5)	1	Ref	–	1	Ref	–
Yes	580 (40.6)	0.96	(0.78, 1.19)	0.74	0.99	(0.78, 1.26)	0.93
**Sexual identity**							
Gay/Homosexual	633 (41.3)	1	Ref	–	1	Ref	–
Bisexual	104 (41.4)	1.01	(0.77, 1.32)	0.96	**1.74**	**(1.22, 2.48)**	**0.002**
Other †	39 (34.5)	0.75	(0.50, 1.12)	0.16	1.02	(0.61, 1.68)	0.95
**Outness**							
None	29 (22.7)	1	Ref	–	1	Ref	–
Less than half	165 (33.9)	**1.75**	**(1.11, 2.76)**	**0.02**	**1.72**	**(1.02, 2.91)**	**0.04**
More than half	143 (43.5)	**2.62**	**(1.64, 4.19)**	**<0.001**	**2.29**	**(1.31, 4.00)**	**0.004**
All or almost all	431 (46.6)	**2.98**	**(1.93, 4.60)**	**<0.001**	**3.20**	**(1.88, 5.46)**	**<0.001**
**Currently using PrEP**							
No	724 (40.0)	1	Ref	–	1	Ref	–
Yes	45 (59.2)	**2.18**	**(1.36, 3.47)**	**0.001**	1.34	(0.76, 2.36)	0.31
**HIV testing history**							
Never tested	152 (33.9)	1	Ref	–	1	Ref	–
Last HIV test negative	620 (43.1)	**1.48**	**(1.18, 1.84)**	**0.001**	1.25	(0.94, 1.66)	0.12
**CAI in last 12 months**							
0	388 (34.3)	1	Ref	–	1	Ref	–
1–2	187 (50.7)	**1.97**	**(1.55, 2.50)**	**<0.001**	**1.79**	**(1.34, 2.38)**	**<0.001**
3–5	77 (46.7)	**1.68**	**(1.21, 2.33)**	**0.002**	1.45	(0.99, 2.12)	0.06
6+	89 (56.3)	**2.47**	**(1.76, 3.47)**	**<0.001**	**1.79**	**(1.18, 2.71)**	**0.01**
**Last sex encounter was group sex**							
No	506 (44.5)	1	Ref	–	1	Ref	–
Yes	103 (44.0)	0.98	(0.74, 1.30)	0.88	0.97	(0.70, 1.34)	0.84
*Missing ^*	*167 (31*.*6)*	***0*.*58***	***(0*.*46*, *0*.*72)***	***<0*.*001***	*0*.*82*	*(0*.*62*, *1*.*08)*	*0*.*16*
**STI in last 12 months**							
No	603 (37.9)	1	Ref	–	1	Ref	–
Yes	145 (57.5)	**2.22**	**(1.69, 2.90)**	**<0.001**	**1.53**	**(1.12, 2.10)**	**0.01**
**Anxiety/Depression**							
None	284 (35.1)	1	Ref	–	1	Ref	–
Mild	300 (45.5)	**1.55**	**(1.25, 1.91)**	**<0.001**	**1.45**	**(1.14, 1.84)**	**0.002**
Moderate	103 (44.4)	**1.48**	**(1.10, 1.99)**	**0.01**	1.07	(0.76, 1.51)	0.69
Severe	81 (46.8)	**1.63**	**(1.17, 2.27)**	**0.004**	1.29	(0.88, 1.89)	0.19

*: All independent covariates included in the model are adjusted for one another.

^: Indicator variables were synthesised for categorical variables with ≥5% missing data.

+: Retired, on long-term sick leave, or other.

†: All other terms, or does not use a term.

GbMSM = Gay, bisexual, and other men who have sex with men; HIV = Human immunodeficiency virus; EMIS = European MSM Internet Survey; AOR = Adjusted odds ratio; 95% CI = 95% confidence interval; RDU = Recreational drug use; PrEP = Pre-exposure prophylaxis; CAI = Condomless anal intercourse; STI = Sexually transmitted infection.

#### 3.1.2. SDU among gbMSM without a diagnosis of HIV

As shown in Table 4, gbMSM who engaged in SDU in the previous year were more likely to self-identify as bisexual (vs. gay/homosexual) (AOR 1.75, 95% CI 1.05–2.92), report any number of non-steady CAI partners in the previous year (vs. none) (1–2 partners; AOR 3.16, 95% CI 2.05–4.88, 3–5 partners; AOR 2.50, 95% CI 1.47–4.26, 6+ partners; AOR 3.79, 95% CI 2.23–6.43), self-report a bacterial STI diagnosis within the same timeframe (vs. none) (AOR 2.15, 95% CI 1.48–3.12), and live in Dublin (vs. outside Dublin) (AOR 1.50, 95% CI 1.07–2.10). Furthermore, the odds of SDU were significantly higher among gbMSM who reported group sex in their last sexual encounter (vs. none) (AOR 1.73, 95% CI 1.15–2.60), and in gbMSM who had attended for HIV testing but had a negative test result (vs. never tested) (AOR 2.02, 95% CI 1.20–3.39). GbMSM who were students were less likely to report SDU (vs. employed gbMSM) (AOR 0.50, 95% CI 0.29–0.86).

**Table 4:** Factors associated with engagement in sexualised drug use in the previous 12 months among gbMSM without a diagnosis of HIV who took part in EMIS-2017 and were included in the study sample (n = 1,898).

	SDU n (%)	OR	Univariable analysis 95% CI	P-Value	AOR	Multivariable analysis 95% CI	P-Value
**Age**							
40+	61 (10.8)	1	Ref	–	1	Ref	–
25–39	138 (15.5)	**1.52**	**(1.10, 2.10)**	**0.01**	1.30	(0.89, 1.92)	0.18
18–24	50 (11.3)	1.05	(0.71, 1.57)	0.80	1.62	(0.95, 2.78)	0.08
**Education >16 years**							
0–3 years	47 (12.4)	1	Ref	–	1	Ref	–
4–6 years	106 (14.5)	1.21	(0.83, 1.74)	0.32	1.07	(0.69, 1.64)	0.77
+7 years	70 (11.4)	0.91	(0.61, 1.35)	0.64	0.85	(0.54, 1.35)	0.49
*Missing* ^	*26 (14*.*9)*	*1*.*24*	*(0*.*74*, *2*.*09)*	*0*.*41*	*1*.*11*	*(0*.*60*, *2*.*07)*	*0*.*74*
**Employment**							
Employed	202 (14.6)	1	Ref	–	1	Ref	–
Unemployed	10 (11.9)	0.79	(0.40, 1.55)	0.49	1.15	(0.55, 2.41)	0.70
Student	30 (8.7)	**0.55**	**(0.37, 0.83)**	**0.004**	**0.50**	**(0.29, 0.86)**	**0.01**
Other +	5 (6.0)	**0.37**	**(0.15, 0.93)**	**0.04**	0.29	(0.08, 1.00)	0.05
**Area of residence**							
Outside Dublin	76 (9.8)	1	Ref	–	1	Ref	–
Dublin	159 (15.7)	**1.72**	**(1.29, 2.30)**	**<0.001**	**1.50**	**(1.07, 2.10)**	**0.02**
*Missing* ^	*14 (12*.*7)*	*1*.*35*	(0.73, 2.47)	*0*.*34*	*1*.*30*	(0.64, 2.62)	*0*.*47*
**Born in Ireland**							
No	64 (13.8)	1	Ref	–	1	Ref	–
Yes	184 (12.9)	0.93	(0.68, 1.26)	0.63	1.02	(0.72, 1.45)	0.90
**Sexual identity**							
Gay/Homosexual	208 (13.6)	1	Ref	–	1	Ref	–
Bisexual	31 (12.4)	0.90	(0.60, 1.34)	0.60	**1.75**	**(1.05, 2.92)**	**0.03**
Other †	10 (8.8)	0.62	(0.32, 1.20)	0.16	0.73	(0.31, 1.71)	0.47
**Outness**							
None	10 (7.8)	1	Ref	–	1	Ref	–
Less than half	61 (12.5)	1.69	(0.84, 3.40)	0.14	1.86	(0.77, 4.50)	0.17
More than half	41 (12.5)	1.68	(0.81, 3.46)	0.16	1.41	(0.55, 3.62)	0.47
All or almost all	136 (14.7)	**2.04**	**(1.04, 3.98)**	**0.04**	1.76	(0.72, 4.31)	0.22
**Currently using PrEP**							
No	220 (12.2)	1	Ref	–	1	Ref	–
Yes	25 (32.9)	**3.54**	**(2.15, 5.83)**	**<0.001**	1.50	(0.81, 2.80)	0.20
**HIV testing history**							
Never tested	29 (6.5)	1	Ref	–	1	Ref	–
Last HIV test negative	219 (15.2)	**2.60**	**(1.73, 3.88)**	**<0.001**	**2.02**	**(1.20, 3.39)**	**0.01**
**CAI in last 12 months**							
0	83 (7.3)	1	Ref	–	1	Ref	–
1–2	71 (19.2)	**3.01**	**(2.14, 4.24)**	**<0.001**	**3.16**	**(2.05, 4.88)**	**<0.001**
3–5	33 (20.0)	**3.16**	**(2.03, 4.92)**	**<0.001**	**2.50**	**(1.47, 4.26)**	**0.001**
6+	51 (32.3)	**6.02**	**(4.03, 9.00)**	**<0.001**	**3.79**	**(2.23, 6.43)**	**<0.001**
**Last sex encounter was group sex**							
No	152 (13.4)	1	Ref	–	1	Ref	–
Yes	54 (23.1)	**1.94**	**(1.37, 2.75)**	**<0.001**	**1.73**	**(1.15, 2.60)**	**0.01**
***Missing ^***	***43 (8*.*1)***	***0*.*57***	***(0*.*40*, *0*.*82)***	***0*.*002***	***1*.*56***	***(0*.*96*, *2*.*52)***	***0*.*07***
**STI in last 12 months**							
No	164 (10.3)	1	Ref	–	1	Ref	–
Yes	76 (30.2)	**3.75**	**(2.74, 5.14)**	**<0.001**	**2.15**	**(1.48, 3.12)**	**<0.001**
**Anxiety/Depression**							
None	98 (12.1)	1	Ref	–	1	Ref	–
Mild	86 (13.1)	1.09	(0.80, 1.49)	0.58	1.11	(0.78, 1.58)	0.56
Moderate	42 (18.1)	**1.61**	**(1.08, 2.38)**	**0.02**	1.55	(0.98, 2.45)	0.06
Severe	19 (11.0)	0.90	(0.53, 1.51)	0.68	0.86	(0.47, 1.58)	0.62

*: All independent covariates included in the model are adjusted for one another.

^: Indicator variables were synthesised for categorical variables with ≥5% missing data.

+: Retired, on long-term sick leave, or other.

†: All other terms, or does not use a term.

GbMSM = Gay, bisexual, and other men who have sex with men; HIV = Human immunodeficiency virus; EMIS = European MSM Internet Survey; AOR = Adjusted odds ratio; 95% CI = 95% confidence interval; RDU = Recreational drug use; PrEP = Pre-exposure prophylaxis; CAI = Condomless anal intercourse; STI = Sexually transmitted infection.

#### 3.1.3. RDU among gbMSM with diagnosis of HIV

In Table 5, higher odds of RDU were found among HIV-diagnosed gbMSM who screened positive for severe anxiety/depression (vs. none) (AOR 5.86, 95% CI 1.21–28.37), and among those who disclosed their sexual orientation to all or almost all of their family/close friends (vs. less than half) (AOR 5.69, 95% CI 1.38–23.56).

**Table 5:** Factors associated with engagement in recreational drug use (excluding poppers) in the previous 12 months among gbMSM with a diagnosis of HIV who took part in EMIS-2017 and were included in the study sample (n = 141).

	RDU n (%)	OR	Univariable analysis 95% CI	P-Value	AOR	Multivariable analysis 95% CI	P-Value
**Age**							
40+	33 (46.5)	1	Ref	–	1	Ref	–
25–39	38 (56.7)	1.51	(0.77, 2.95)	0.23	2.88	(0.02, 8.24)	0.08
18–24	2 (66.7)	2.30	(0.20, 26.56)	0.50	0.37	(0.02, 8.36)	0.53
**Education >16 years**							
0–3 years	11 (39.3)	1	Ref	–	1	Ref	–
4–6 years	23 (45.1)	1.27	(0.50, 3.24)	0.62	0.71	(0.17, 2.97)	0.64
+7 years	34 (66.7)	**3.09**	**(1.19, 8.04)**	**0.02**	2.89	(0.70, 11.89)	0.14
*Missing* ^	*5 (45*.*5)*	*1*.*29*	*(0*.*31*, *5*.*27)*	*0*.*73*	*0*.*47*	*(0*.*06*, *3*.*99)*	*0*.*49*
**Employment**							
Employed	58 (52.7)	1	Ref	–	1	Ref	–
Unemployed	4 (50.0)	0.90	(0.21, 3.77)	0.88	1.38	(0.08, 23.60)	0.82
Student	3 (60.0)	1.34	(0.22, 8.37)	0.75	5.29	(0.36, 78.64)	0.23
Other +	6 (37.5)	0.54	(0.18, 1.58)	0.26	0.75	(0.11, 4.90)	0.76
**Area of residence**							
Outside Dublin	19 (38.0)	1	Ref	–	1	Ref	–
Dublin	48 (57.8)	**2.24**	**(1.09, 4.59)**	**0.03**	2.03	(0.74, 5.57)	0.17
**Born in Ireland**							
No	26 (59.1)	1	Ref	–	1	Ref	–
Yes	47 (48.5)	0.65	(0.32, 1.34)	0.24	0.88	(0.30, 2.61)	0.82
**Sexual identity**							
Gay/Homosexual	69 (55.2)	1	Ref	–	1	Ref	–
Bisexual	0 (0.0)	–	–	–	–	–	–
Other †	4 (40.0)	0.54	(0.15, 2.01)	0.36	0.71	(0.07, 7.13)	0.77
**Outness**							
None	0 (0.0)	–	–	–	–	–	–
Less than half	8 (28.6)	1	Ref	–	1	Ref	–
More than half	11 (61.1)	**3.93**	**(1.12, 13.75)**	**0.03**	4.07	(0.48, 34.48)	0.20
All or almost all	54 (58.1)	**3.46**	**(1.38, 8.66)**	**0.01**	**5.69**	**(1.38, 23.56)**	**0.02**
**CAI in last 12 months**							
0	15 (36.6)	1	Ref	–	1	Ref	–
1–2	10 (40.0)	1.16	(0.42, 3.21)	0.78	1.33	(0.24, 7.33)	0.74
3–5	9 (47.4)	1.56	(0.52, 4.70)	0.43	0.83	(0.13, 5.30)	0.85
6+	31 (68.9)	**3.84**	**(1.57, 9.40)**	**0.003**	2.75	(0.50, 15.07)	0.24
**Last sex encounter was group sex**							
No	37 (49.3)	1	Ref	–	1	Ref	–
Yes	26 (66.7)	2.05	(0.92, 4.59)	0.08	2.08	(0.56, 7.68)	0.27
*Missing* ^	*10 (37*.*0)*	*0*.*60*	*(0*.*24*, *1*.*49)*	*0*.*27*	*0*.*53*	*(0*.*09*, *3*.*00)*	*0*.*47*
**STI in last 12 months**							
No	49 (50.0)	1	Ref	–	1	Ref	–
Yes	23 (60.5)	1.53	(0.72, 3.28)	0.27	1.18	(0.37, 3.80)	0.78
**Anxiety/Depression**							
None	28 (50.0)	1	Ref	–	1	Ref	–
Mild	26 (53.1)	1.13	(0.52, 2.44)	0.75	0.94	(0.31, 2.86)	0.92
Moderate	7 (46.7)	0.88	(0.28, 2.74)	0.82	1.53	(0.27, 8.86)	0.63
Severe	12 (63.2)	1.71	(0.59, 4.99)	0.32	**5.86**	**(1.21, 28.37)**	**0.03**

*: All independent covariates included in the model are adjusted for one another.

^: Indicator variables were synthesised for categorical variables with ≥5% missing data.

+: Retired, on long-term sick leave, or other.

†: All other terms, or does not use a term.

GbMSM = Gay, bisexual, and other men who have sex with men; HIV = Human immunodeficiency virus; EMIS = European MSM Internet Survey; AOR = Adjusted odds ratio; 95% CI = 95% confidence interval; RDU = Recreational drug use; CAI = Condomless anal intercourse; STI = Sexually transmitted infection.

#### 3.1.4. SDU among gbMSM with a diagnosis of HIV

Finally, in Table 6, greater odds of SDU were found among gbMSM with a positive diagnosis of HIV who resided in Dublin (vs. outside Dublin) (AOR 5.00, 95% CI 1.13–22.12), and among those who reported 6+ non-steady CAI partners in the previous year (vs. none) (AOR 17.42, 95% CI 1.37–221.78).

**Table 6:** Factors associated with engagement in sexualised drug use in the previous 12 months among gbMSM with a diagnosis of HIV who took part in EMIS-2017 and were included in the study sample (n = 141).

	SDU n (%)	OR	Univariable analysis 95% CI	P-Value	AOR	Multivariable analysis 95% CI	P-Value
**Age**							
40+	18 (25.4)	1	Ref	–	1	Ref	–
25–39	18 (26.9)	1.08	(0.51, 2.31)	0.84	1.05	(0.26, 4.20)	0.94
18–24	1 (33.3)	1.47	(0.13, 17.22)	0.76	0.18	(0.004, 7.51)	0.36
**Education >16 years**							
0–3 years	6 (21.4)	1	Ref	–	1	Ref	–
4–6 years	11 (21.6)	1.01	(0.33, 3.10)	0.99	0.72	(0.12, 4.28)	0.72
+7 years	17 (33.3)	1.83	(0.63, 5.37)	0.27	3.00	(0.54, 16.51)	0.21
*Missing* ^	*3 (27*.*3)*	*1*.*38*	*(0*.*28*, *6*.*84)*	*0*.*70*	*0*.*30*	*(0*.*02*, *5*.*62)*	*0*.*42*
**Employment**							
Employed	32 (29.1)	1	Ref	–	1	Ref	–
Unemployed	3 (37.5)	1.46	(0.33, 6.48)	0.62	26.55	(0.82, 856.46)	0.06
Student	1 (20.0)	0.61	(0.07, 5.66)	0.66	9.48	(0.34, 263.03)	0.19
Other +	1 (6.3)	0.16	(0.02, 1.28)	0.09	0.08	(0.002, 2.60)	0.16
**Area of residence**							
Outside Dublin	6 (12.0)	1	Ref	–	1	Ref	–
Dublin	25 (30.1)	**3.16**	**(1.19, 8.37)**	**0.02**	**5.00**	**(1.13, 22.12)**	**0.03**
**Born in Ireland**							
No	15 (34.1)	1	Ref	–	1	Ref	–
Yes	22 (22.7)	0.57	(0.26, 1.24)	0.16	0.72	(0.19, 2.73)	0.63
**Sexual identity**							
Gay/Homosexual	36 (28.8)	1	Ref	–	1	Ref	–
Bisexual	0 (0.0)	–	–	–	–	–	–
Other †	1 (10.0)	0.27	(0.03, 2.25)	0.23	0.19	(0.01, 3.40)	0.26
**Outness**							
None	0 (0.0)	–	–	–	–	–	–
Less than half	4 (14.3)	1	Ref	–	1	Ref	–
More than half	6 (33.3)	3.00	(0.71, 12.69)	0.14	1.58	(0.12, 19.96)	0.73
All or almost all	27 (29.0)	2.45	(0.78, 7.75)	0.13	1.60	(0.28, 9.18)	0.60
**CAI in last 12 months**							
0	2 (4.9)	1	Ref	–	1	Ref	–
1–2	4 (16.0)	3.71	(0.63, 21.99)	0.15	4.85	(0.34, 69.81)	0.25
3–5	7 (36.8)	**11.38**	**(2.08, 62.23)**	**0.01**	2.87	(0.17, 47.38)	0.46
6+	22 (48.9)	**18.65**	**(4.01, 86.70)**	**<0.001**	**17.42**	**(1.37, 221.78)**	**0.03**
**Last sex encounter was group sex**							
No	18 (24.0)	1	Ref	–	1	Ref	–
Yes	17 (43.6)	**2.45**	**(1.07, 5.59)**	**0.03**	2.45	(0.61, 9.81)	0.20
*Missing* ^	*2 (7*.*4)*	*0*.*25*	*(0*.*05*, *1*.*18)*	*0*.*08*	*1*.*04*	*(0*.*05*, *22*.*70)*	*0*.*98*
**STI in last 12 months**							
No	23 (23.5)	1	Ref	–	1	Ref	–
Yes	13 (34.2)	1.70	(0.75, 3.84)	0.21	0.56	(0.13, 2.32)	0.42
**Anxiety/Depression**							
None	15 (26.8)	1	Ref	–	1	Ref	–
Mild	13 (26.5)	0.99	(0.41, 2.35)	0.98	0.61	(0.17, 2.23)	0.46
Moderate	5 (33.3)	1.37	(0.40, 4.66)	0.62	1.51	(0.18, 12.76)	0.70
Severe	4 (21.1)	0.73	(0.21, 2.55)	0.62	2.36	(0.27, 20.27)	0.44

*: All independent covariates included in the model are adjusted for one another.

^: Indicator variables were synthesised for categorical variables with ≥5% missing data.

+: Retired, on long-term sick leave, or other.

†: All other terms, or does not use a term

GbMSM = Gay, bisexual, and other men who have sex with men; HIV = Human immunodeficiency virus; EMIS = European MSM Internet Survey; AOR = Adjusted odds ratio; 95% CI = 95% confidence interval; RDU = Recreational drug use; CAI = Condomless anal intercourse; STI = Sexually transmitted infection.

## 4. Discussion

### 4.1. Principal findings

GbMSM in our sample reported a high prevalence of past-year RDU (51.8% and 40.9% among those with and without a diagnosis of HIV, respectively). These estimates are in stark contrast to the 2019/2020 prevalence estimates of RDU among the general male population in Ireland (12.3%) [7]. Notably, our analysis was stratified based on HIV status because previous literature has suggested that HIV-positive gbMSM can systematically differ from HIV-negative men in certain aspects. For example, HIV-positive gbMSM are typically more likely to engage in substance use [19, 46]. They also tend to have a high level of formal engagement with healthcare services due to their diagnosis which, by default, presents opportunities for health promotion and preventive messaging from trusted healthcare professionals [47]. Finally, current PrEP use (an independent covariate included in our study) was specifically used by non-diagnosed positive gbMSM only. Consequently, stratification by HIV status was required to prevent the introduction of systematic error in our multivariable models.

Among gbMSM without a prior diagnosis of HIV, those who were younger were more likely to engage in RDU in the previous year. GbMSM who resided in Dublin were almost one and a half times more likely to report RDU. These findings are consistent with the results of prior studies [30, 39]. Among the general male population in Ireland, 15–24 year olds represent the age category with the highest burden of past-year drug use [6, 7], and our study revealed a similar pattern (albeit amongst 18–24 year olds). Conversely, while previous research suggests that SDU (specifically chemsex) is most common in the late twenties, thirties, and early forties among gbMSM [21, 37], our study did not elucidate any significant relationship between age and SDU. However, EMIS-2017 omitted GHB/GBL from its definition of SDU, and included a number of other drugs, such as cocaine and MDMA. Both of these drugs are common at the population-level, particularly among those aged 15–24 years [6], and perhaps their inclusion may have attenuated our findings towards null. Furthermore, gbMSM residing in Dublin had a greater tendency to engage in SDU, irrespective of HIV status, which is consistent with the most recent national study of Irish gbMSM, which analysed data from 2015 [30]. SDU often occurs in private homes, and can be facilitated through smart-phone geospatial networking applications. Therefore, in urban centres such as Dublin, large groups of gbMSM may congregate at relatively short notice [25]. This could potentially account for the higher odds of SDU among respondents who were residing in the capital city.

We observed increased odds of both RDU and SDU among non-diagnosed positive gbMSM who reported CAI encounters with numerous non-steady male partners in the previous 12 months. Non-diagnosed positive gbMSM who self-reported a bacterial STI diagnosis within the same timeframe were 53% and 115% more likely to engage in recent RDU and SDU, respectively. In addition, those who reported that their last sexual encounter involved group sex had a 73% increased odds of engaging in SDU within the previous 12 months. In Ireland, gbMSM account for less than 10% of the overall male population [48], yet it is estimated that they make up 86% and 65% of all incident diagnoses of syphilis and gonorrhoea, respectively [49, 50]. Non-diagnosed gbMSM who presented for HIV testing (and tested negative) were approximately twice as likely to engage in SDU in the previous year. These findings are consistent with previous studies [19, 22, 30, 51, 52]. Indeed, it is beneficial that these men are more proactive about their sexual health by engaging with healthcare services. Furthermore, among HIV-positive gbMSM in our study, 96% had an undetectable viral load due to treatment with ART. This is an important finding for these individuals in relation to their overall health and wellbeing, and it also reduces the likelihood of onward transmission to HIV-negative gbMSM with whom they may engage in sexual activities (i.e. *undetectable = untransmittable [U = U]*). However, our study found no significant association between current PrEP use and engagement in RDU and/or SDU among gbMSM without a diagnosis of HIV, although only 78 participants reported current PrEP use. Importantly, the provision of PrEP has increased dramatically in Ireland due to the national “HIV PrEP” programme which was rolled-out in November 2019 (i.e. after EMIS-2017 was conducted) [53]. Therefore, further research is warranted in Ireland as PrEP has become more widely available, as it may play a key role in curtailing the incidence of HIV among both gbMSM and the general population. Of note, gbMSM account for over 50% of all incident HIV diagnoses annually in Ireland [54].

Increased odds of RDU were more likely among gbMSM who reported anxiety/depression. Specifically, non-diagnosed positive gbMSM who screened positive for a mild level of adverse mental health were approximately 50% more likely to engage in RDU, while HIV-diagnosed gbMSM who reported a severe level of adverse mental health were almost five times more likely to report SDU in the previous year. The exact reasons which underpin this disparity are likely multifactorial and complex. In 2022, a systematic review explored the determinants of depression among gbMSM living with HIV [55], and the authors concluded that HIV-related stigma likely plays a prominent role in the manifestation of poorer mental health outcomes among HIV-diagnosed gbMSM. Separately, in our study, those who were more open about their sexuality were also more likely to report RDU, wherein a gradient effect was observed (among gbMSM without a diagnosis of HIV). Although prior literature has highlighted similar findings in relation to drug use and adverse mental health outcomes [56, 57], greater disclosure of sexual orientation among gbMSM is usually an indication of self-acceptance and improved psychological adjustment [58]. It is usually the “*rejection reactions”* that are typically associated with drug use [59], and this may have contributed to the association we observe between greater “openness” and drug use among gbMSM in our sample. Conversely, another possible explanation for this association could be the long cultural history of drug use in the LGBT+ community, which may be motivated by the desire to experience pleasure, increase empathy and caring, and enhance community spirit [60]. Thus, the more “out” one is, the more likely they are to socialise in gay bars and immerse themselves in gay culture.

Finally, although we may draw comparisons between the prevalence of SDU among our study sample and in previous studies, it is important to consider the exact definition of SDU used, i.e. which specific set of drugs were included, and in what context. A common definition of SDU (specifically chemsex) refers to the consumption of certain drugs (typically crystal methamphetamine, mephedrone, GHB/GBL, and/or ketamine) by gbMSM, before and/or during sex [20–22]. However, an increasing number of sources now qualify cocaine, other stimulants (e.g. MDMA/ecstasy and amphetamine/speed), and new psychoactive substances (e.g. tryptamines) as “chemsex drugs” [19, 31, 61, 62]. Barrett et al. analysed the latest available data (2015) among a nationally-representative sample of Irish gbMSM, and found that 7% of gbMSM used one or more drugs associated with chemsex in the previous year [30]. However, a number of methodological differences from our study must be noted. Firstly, this study qualified crystal methamphetamine, mephedrone, GHB/GBL, and/or ketamine as drugs associated with chemsex. Additionally, this study only asked about the use of these “chemsex drugs” by gbMSM *in general*, as opposed to their specific consumption by gbMSM before and/or during sexual encounters. By contrast, our study refers to the consumption of MDMA/ecstasy, cocaine, amphetamine/speed, crystal methamphetamine, mephedrone, and/or ketamine *in the sexual setting* specifically. Therefore, caution should be exercised when comparing these prevalence estimates. Notably, using data from 2016, Frankis et al. estimated that among gbMSM who reside in Scotland, Wales, Northern Ireland, or the Republic of Ireland, 27% of those who consume one or more “chemsex drugs” (crystal methamphetamine, mephedrone, GHB/GBL, and/or ketamine) each year do not use these drugs in sexual encounters [21].

### 4.2. Strengths and limitations

This study has a number of strengths. Firstly, participants could elect to take the survey in any one of 33 different languages, which increased participation rates from non-English speaking respondents, thereby reducing selection bias. Latin American gbMSM (who may not speak English) are disproportionately impacted by HIV diagnoses in Ireland in comparison to their Irish contemporaries [54]. However, we could not extract meaningful data pertaining to participants’ ethnicities, apart from whether they were born in Ireland or not. This prevented us from assessing specific ethnic subgroups of interest. Secondly, EMIS-2017 was entirely anonymous, which may have increased the internal validity of the study, particularly in relation to sensitive questions (e.g. drug use, HIV/STI diagnoses, and CAI encounters). Previous studies have concluded that online anonymous surveys are the optimal method for assessing drug use [63, 64], and the reduction in social desirability bias in our study likely boosts the validity of our outcome data. Thirdly, we had relatively complete data on study respondents. Most of the variables in our regression models had <1% missing data. Only three variables had ≥5% missing data, and we synthesised indicator variables to control for this. Fourthly, approximately 70% of participants in the overall European dataset were recruited through social networking apps such as PlanetRomeo, Grindr, and Hornet [34]. In Ireland, this respective figure was only about 40% [35]. This was likely due to large community-led efforts with a concerted focus on recruiting gbMSM through gay bars or cafés, gay saunas, gay community centres, and other social venues [35]. Arguably, this may render our findings more generalisable to the wider gbMSM community in Ireland if it encompasses a wider selection of participants, rather than those who engage in the use of dating/networking apps, who tend to be younger, more technologically literature, and have a greater degree of disclose about their sexual orientation [65, 66]. Lastly, the large number of respondents increased our study’s statistical power. However, the smaller quantity of HIV-positive gbMSM in our sample likely introduced type 2 statistical error, which may have prevented us from detecting numerous significant associations [67].

Several limitations of this study merit discussion. Firstly, deciphering the exact temporal sequence of events is not possible, given our study’s cross-sectional design, which hinders our ability to draw any causal conclusions. As a corollary to this limitation, reverse causality must be considered, particularly for certain associations, such as that between drug use and depression/anxiety. Secondly, all data were self-reported, which may have led to information bias. Thirdly, convenience sampling was utilised in order to boost the participation rate which, in comparison to probability-based sampling, tends to enlist gbMSM with higher levels of drug use [68]. This may have inflated our prevalence estimates. Another significant limitation to our study is the exclusion of GHB/GBL in our definition of SDU. Future surveys (including EMIS) should endeavour to include GHB/GBL in questions which specifically relate to SDU/chemsex. Furthermore, we collapsed categories for certain covariates to circumvent the requirement for a Bonferroni correction, while still aiming to decrease the rate of family-wise error [69]. However, this may have reduced the overall variability within our dataset [70]. Finally, although EMIS data are the latest available data on gbMSM across Europe, the survey was undertaken prior to the global COVID-19 pandemic. Perhaps many socio-environmental changes have occurred in the intervening period, which may warrant further research.

### 4.3. Public health implications

Although gbMSM would likely derive benefit from population-based interventions to curtail the harms associated with drug use, these approaches could be complemented by more targeted interventions, and our study has identified potential subgroups of gbMSM who may be at higher odds of engaging in these drug use practices. For example, those who reside in Dublin appear to be at greater odds of RDU and SDU. Peer outreach workers may play an important role in providing direct information to under-reached individuals about safer use and recovery supports, or may signpost these men to available support services [71].

Additional targeted measures, such as brief intervention training for non-specialist healthcare professionals who work closely with the gbMSM community, may also be warranted. Brief interventions are an integral part of the Irish Health Service’s *Making Every Contact Count (MECC)* initiative, and can easily be conducted in many different clinical settings [72]. Harm reduction campaigns may also be of benefit, whereby a concerted focus is placed on reducing the adverse consequences that can be associated with drug use. For example, men who had a recent diagnosis of a bacterial STI and/or who engage in CAI appear to be at higher odds of RDU and SDU. Although it is beneficial that these men have presented for testing, perhaps more efforts should be made to support healthier drug use choices among this cohort (such as emphasising the importance of condom utilisation), given that these men are already engaging with healthcare services. This approach may be particularly effective in reducing drug-related harms amongst those who would not otherwise seek out substance use services [73].

## 5. Conclusion

In this study, gbMSM in Ireland reported a high prevalence of RDU and SDU. This was particularly true for men who were living in Dublin, presented for HIV testing, engaged in more CAI encounters, and reported a recent diagnosis of a bacterial STI. GbMSM who engaged in RDU were more likely to be younger, be more open about their sexuality, and screen positive for adverse mental health outcomes. Messaging should be tailored to subgroups of gbMSM who are likely to be at increased odds of drug-related harms, and our findings may serve as a guideline to relevant partner organisations who work to promote the overall health and wellbeing of gbMSM nationally.

## Supporting information

S1 Appendix Coding and analysis of the remaining covariates. (DOCX)

S2 Appendix Data Transfer Agreement between LSHTM and UCC. (DOCX)

S3 Appendix Prevalence of past-year use of each specific drug included in the “recreational drug use” variable, by HIV-status. (DOCX)
