# Supplementary material for: Recreational and sexualised drug use among gay, bisexual, and other men who have sex with men (gbMSM) in Ireland–Findings from the European MSM internet survey (EMIS) 2017
Source: PLoS One. 2023 Jul 28;18(7):e0288171. doi: 10.1371/journal.pone.0288171 (PMC10381075; doi:10.1371/journal.pone.0288171)
Supplement: S1 Appendix — (DOCX) [file pone.0288171.s001.docx]

# Appendix A – Coding and analysis of the remaining covariates:

Education level, or years spent in the education system beyond 16 years, was also collected as a continuous covariate, but was analysed categorically. It was recoded into four categories: 0-3 years, 4-6 years, 7+ years, or missing. The justification for these categories was that 0-3 years should encompass gbMSM who finished their education at/before Leaving Certificate (secondary school-leaving) level. GbMSM who completed 4-6 extra years likely progressed to third-level education, and those with 7+ additional years most likely included those who attained the highest degree of education (e.g. post-graduate level). Area of residence (i.e. respondents’ county of residence) was collapsed into categories with three potential options: Dublin, outside Dublin, or missing. Employment status was analysed as a categorical variable, and we collapsed categories into four potential options: employed, unemployed, student, or other. “Other” referred to MSM who were retired, on long-term sick leave (medically retired), or any other option. In relation to country of birth, respondents selected the country within which they were born. From this, we derived a new binary covariate: “born in Ireland” (yes/no). Sexual identity was analysed as a categorical variable, and categories were collapsed here as well, which yielded three potential options: gay/homosexual, bisexual, or other. “Other” referred to straight/homosexual, any other term, or “do not usually use a term”. Respondents’ degree of sexual orientation disclosure, i.e. “outness”, was also analysed as a categorical covariate. This question asked about the number of family/close friends that were aware of participants’ attraction to men, yielding five potential choices: all or almost all, more than half, less than half, few, or none. We then collapsed categories for this variable, which yielded four options: all or almost all, more than half, less than half, or none. A binary variable (yes/no) for bacterial STI diagnosis in the previous 12 months was created, and this included a self-reported diagnosis of syphilis, gonorrhoea, and/or chlamydia (including lymphogranuloma venereum). HIV testing history was collapsed into a binary variable (never tested, or last test negative), and current PrEP use was also analysed as a binary variable (yes/no). Originally, “diagnosed positive” was another potential option, but this was subsequently removed, as all men who received a positive diagnosis for HIV were included in our sensitivity analysis.
