# Supplementary figures and images for: Recreational and sexualised drug use among gay, bisexual, and other men who have sex with men (gbMSM) in Ireland–Findings from the European MSM internet survey (EMIS) 2017
Source: PLoS One. 2023 Jul 28;18(7):e0288171. doi: 10.1371/journal.pone.0288171 (PMC10381075; doi:10.1371/journal.pone.0288171)

# Appendix B – Data Transfer Agreement between LSHTM and UCC:


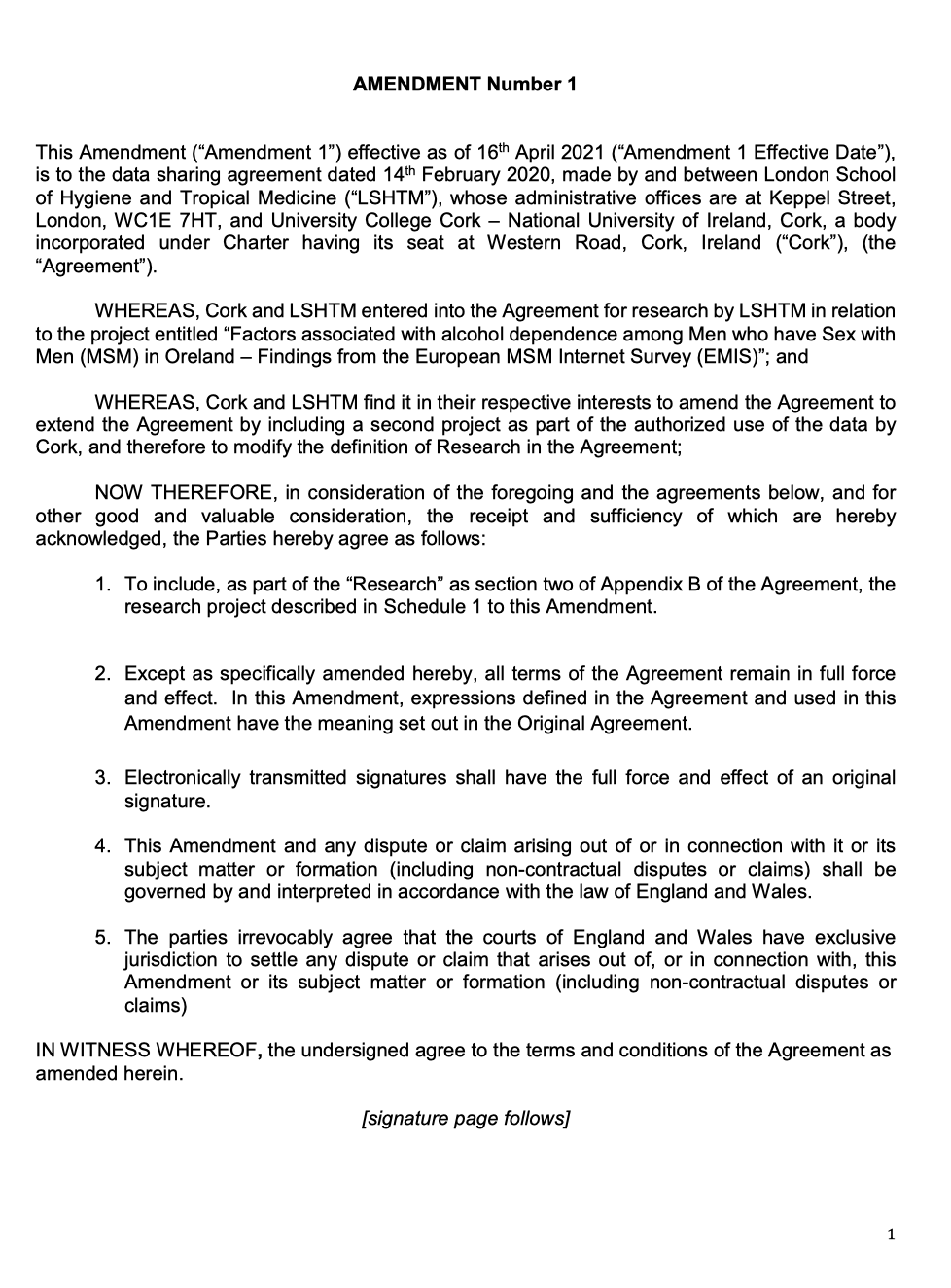


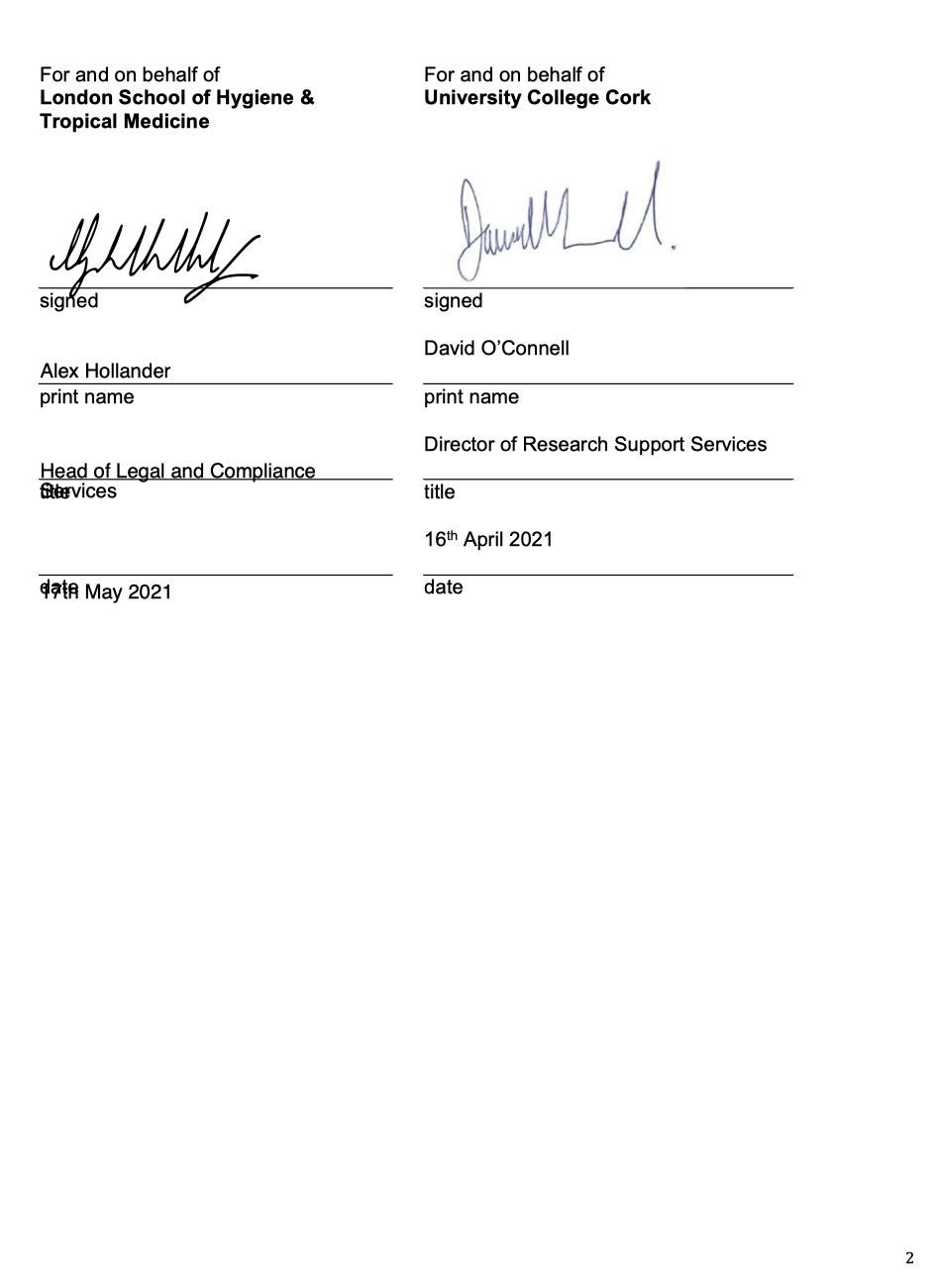


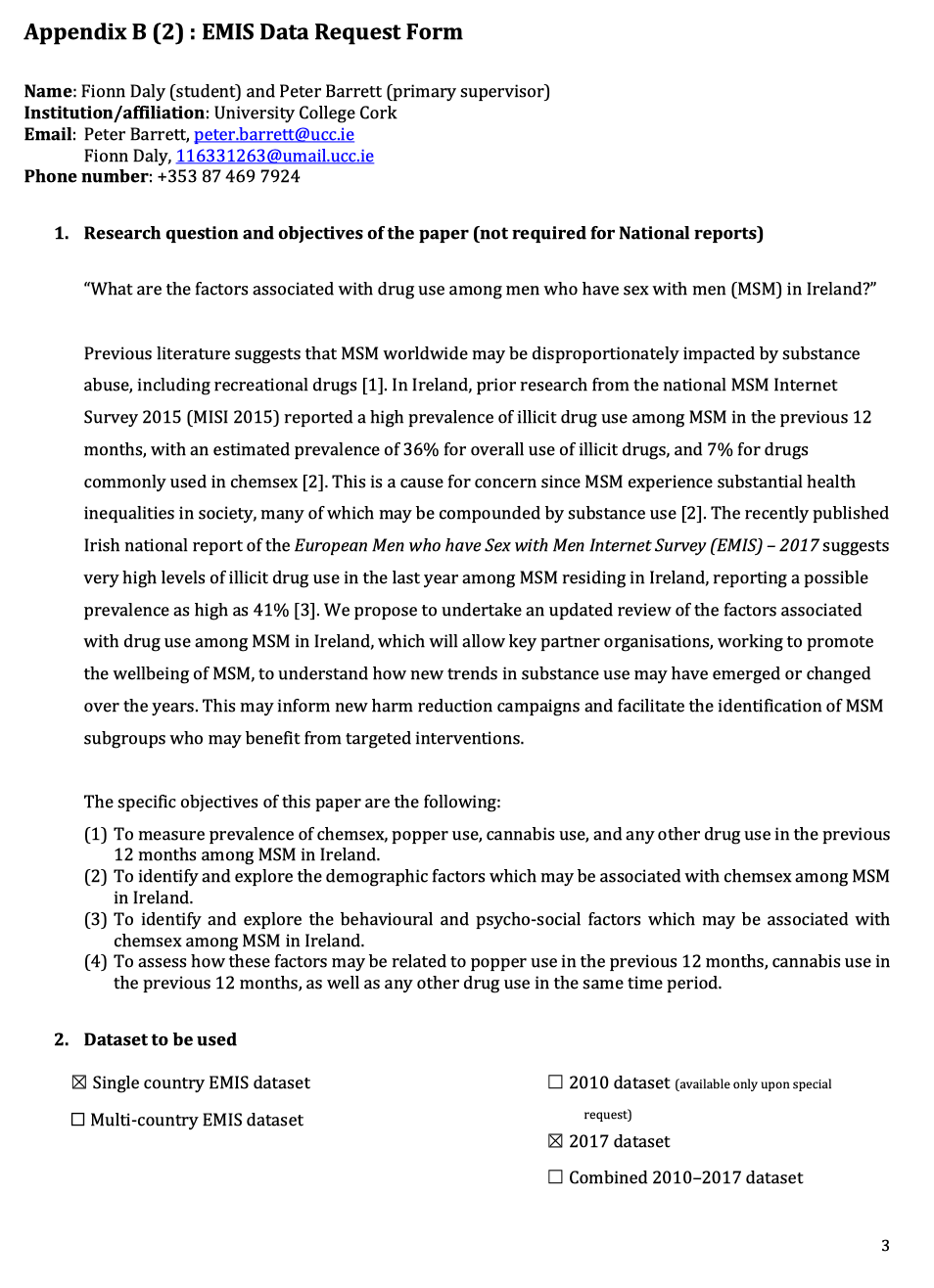


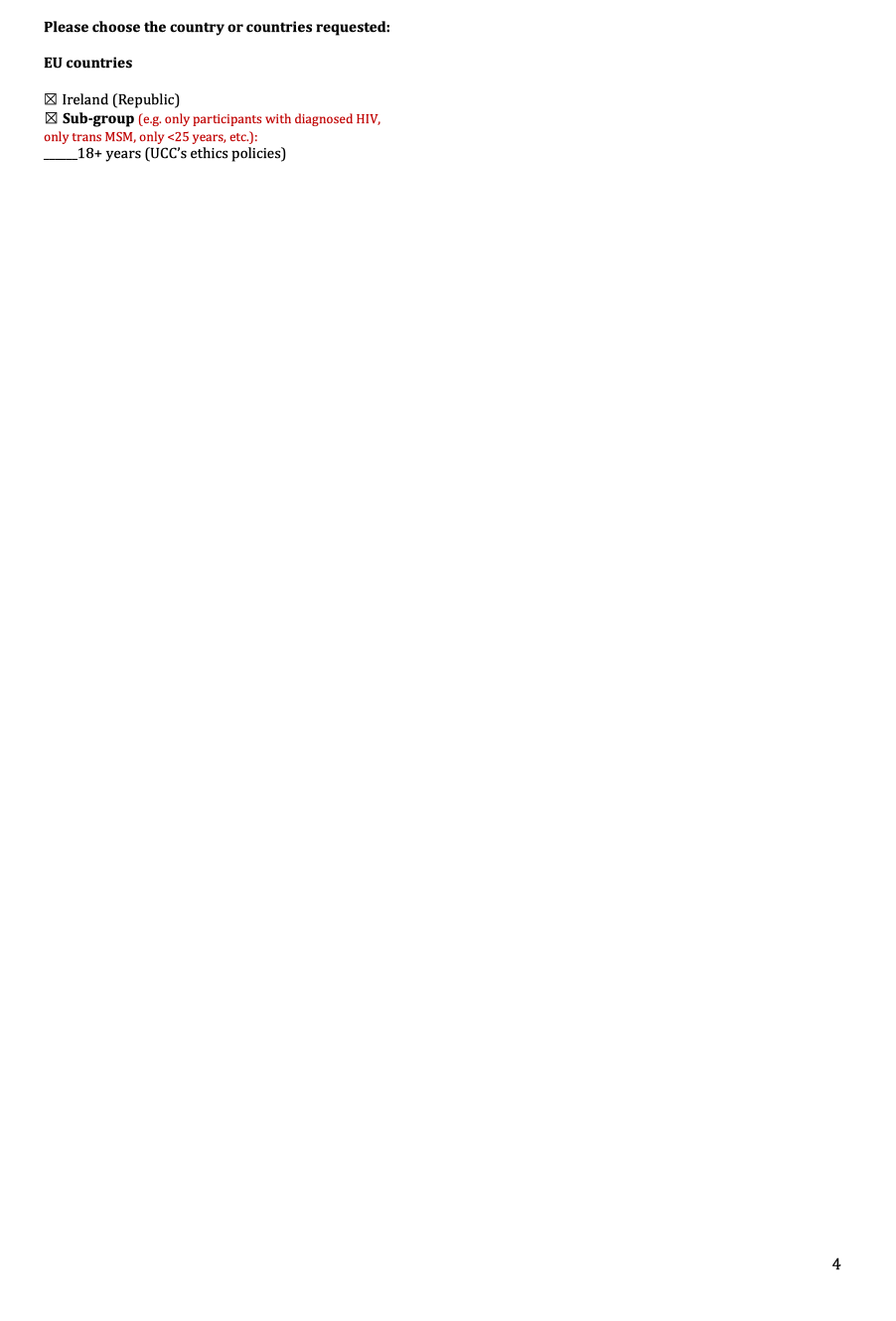


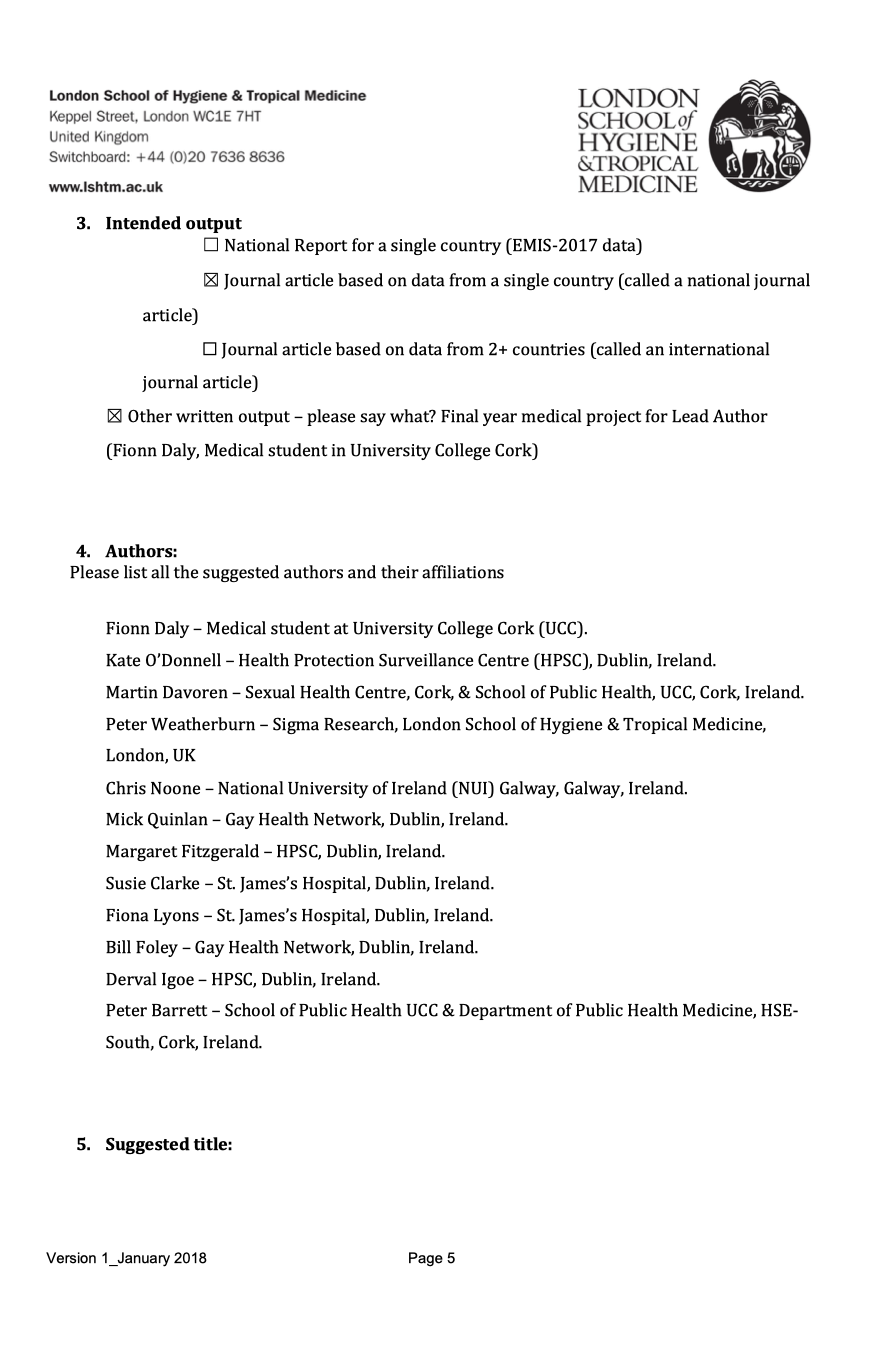


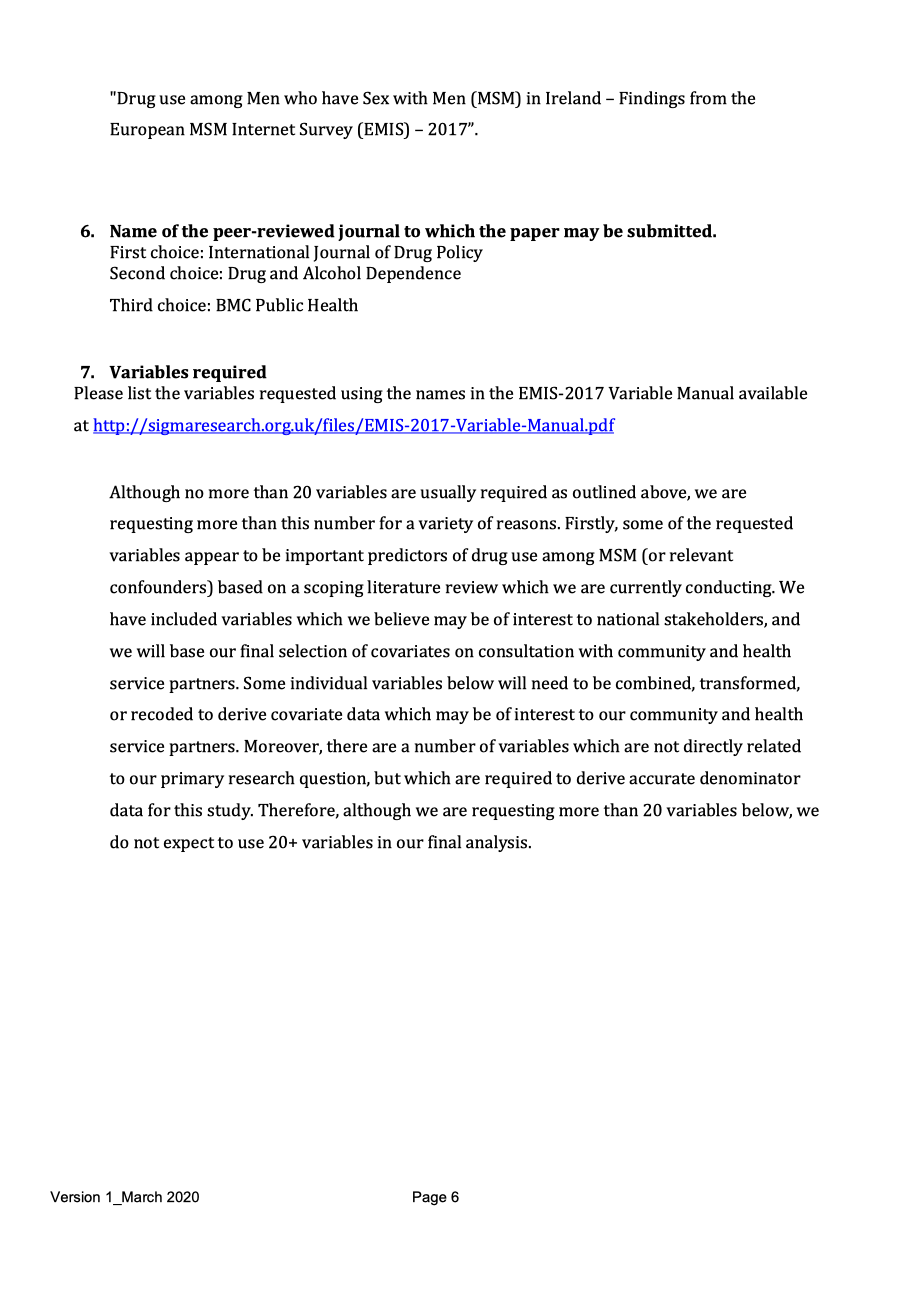


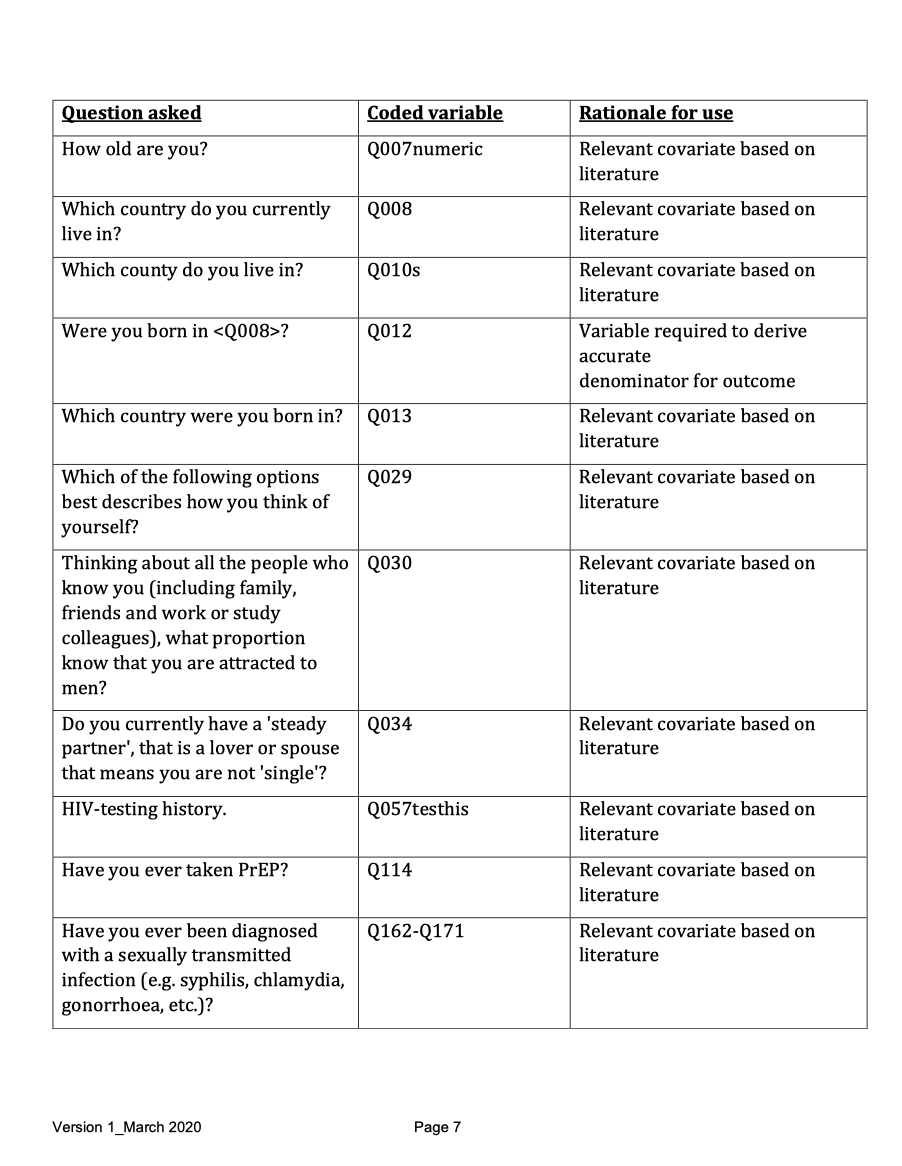


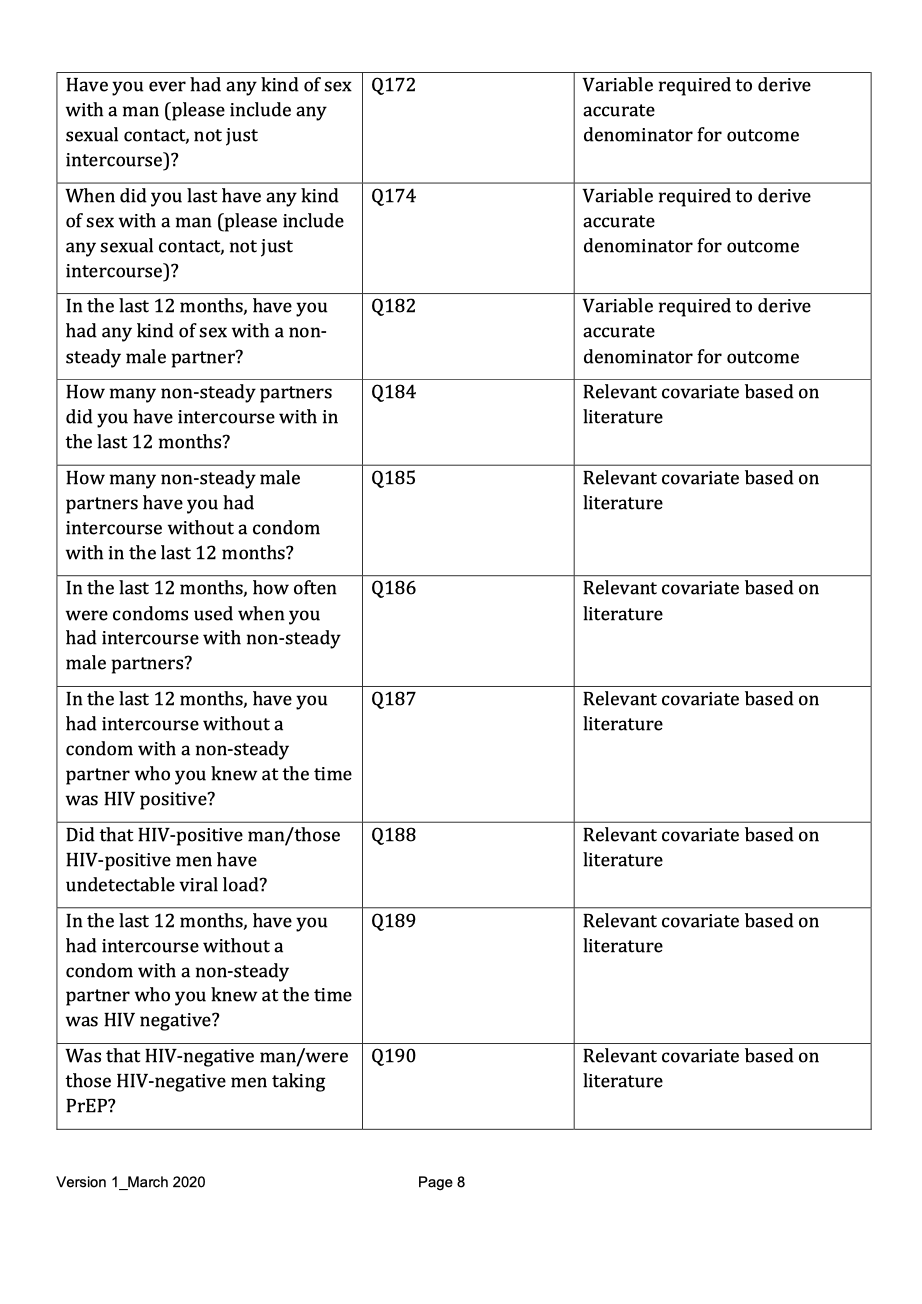


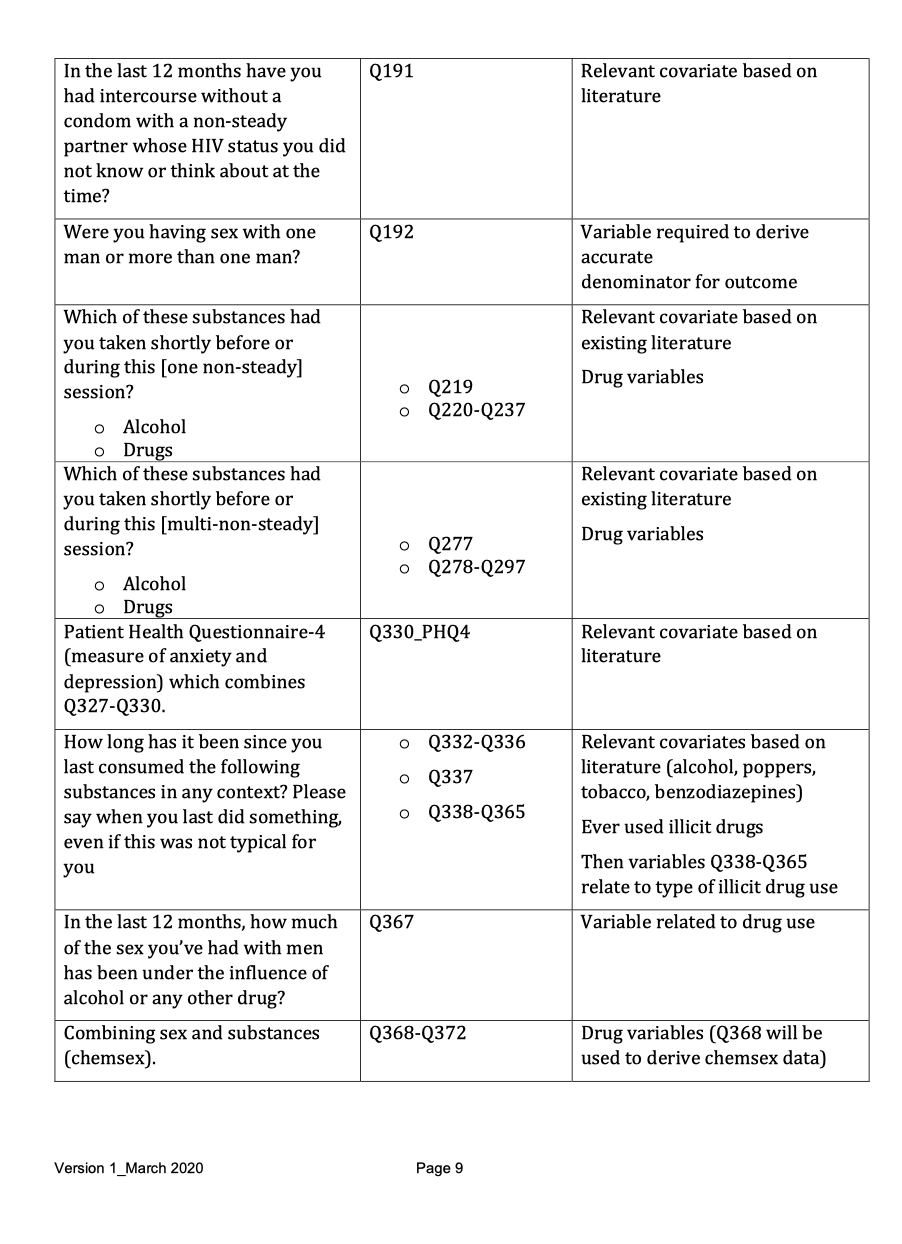


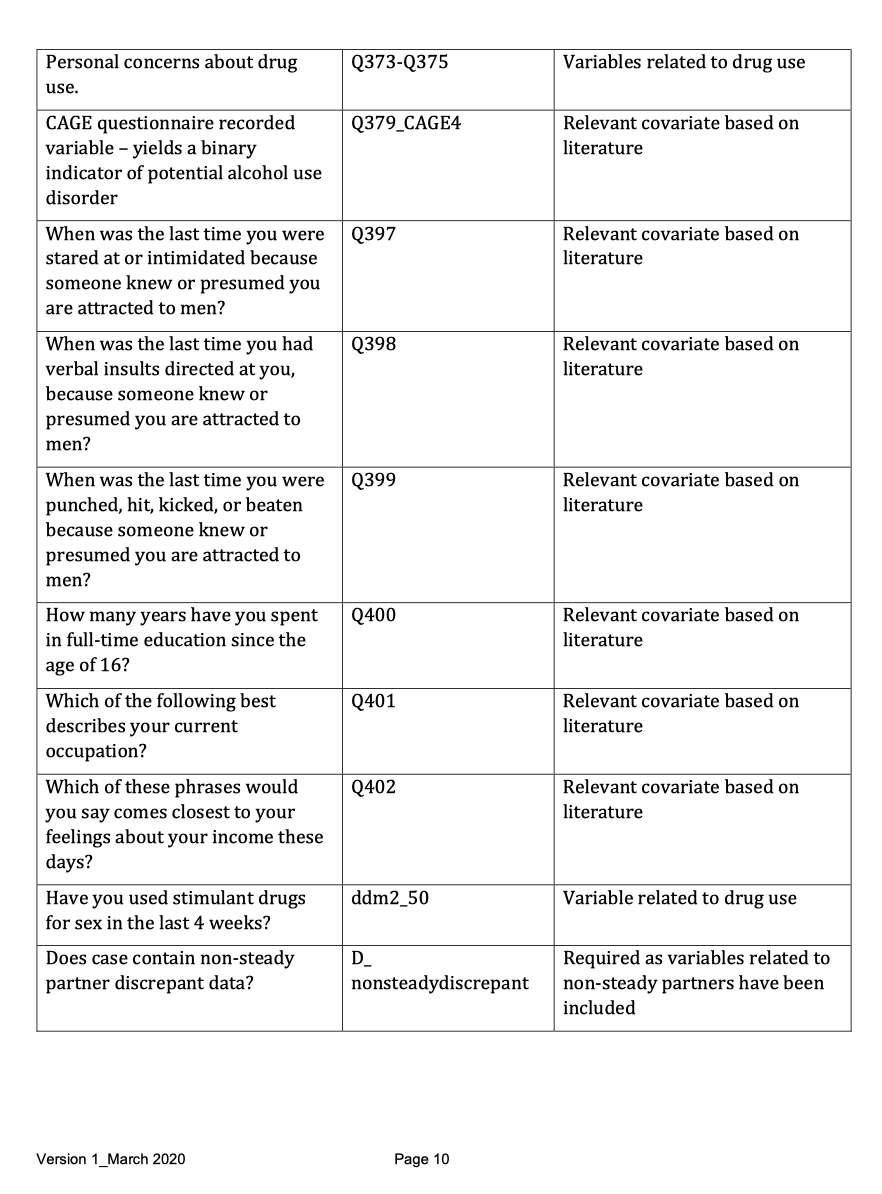


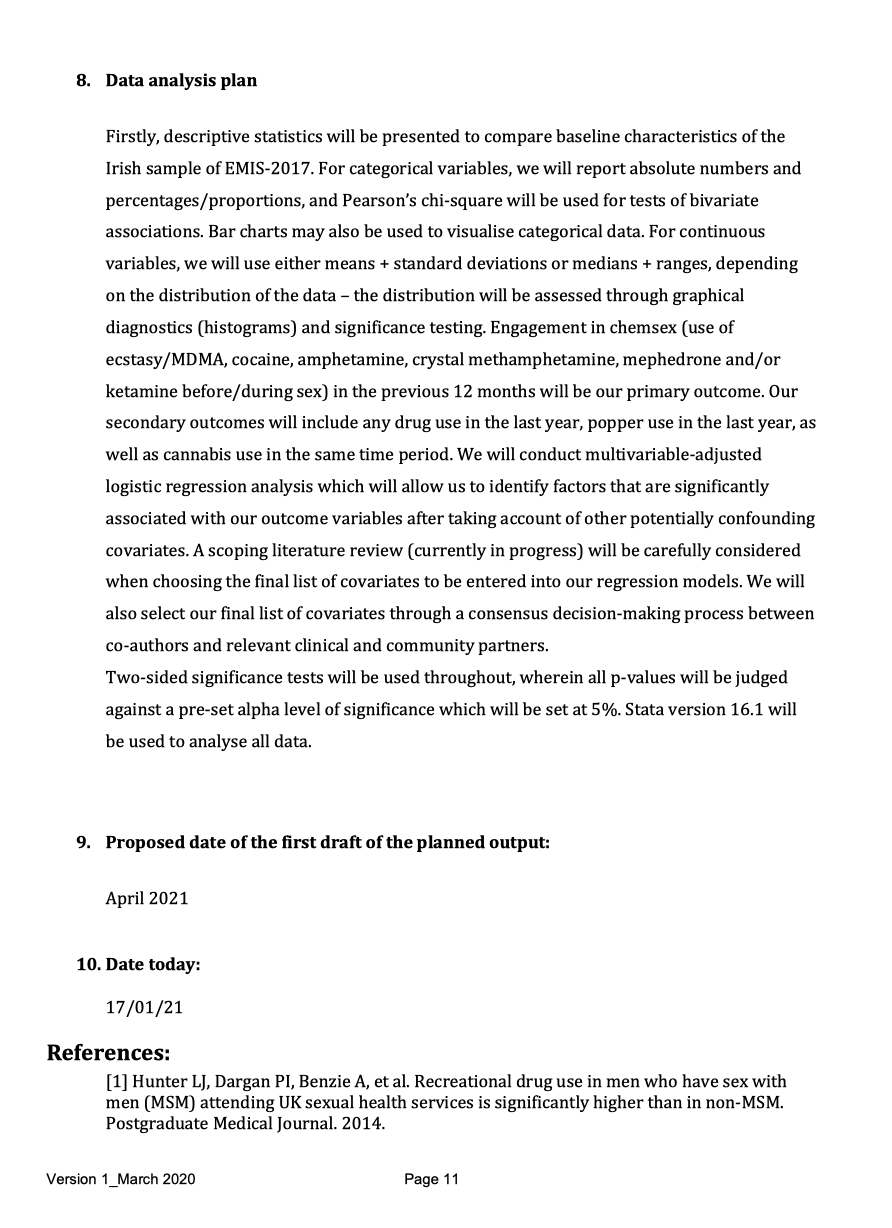


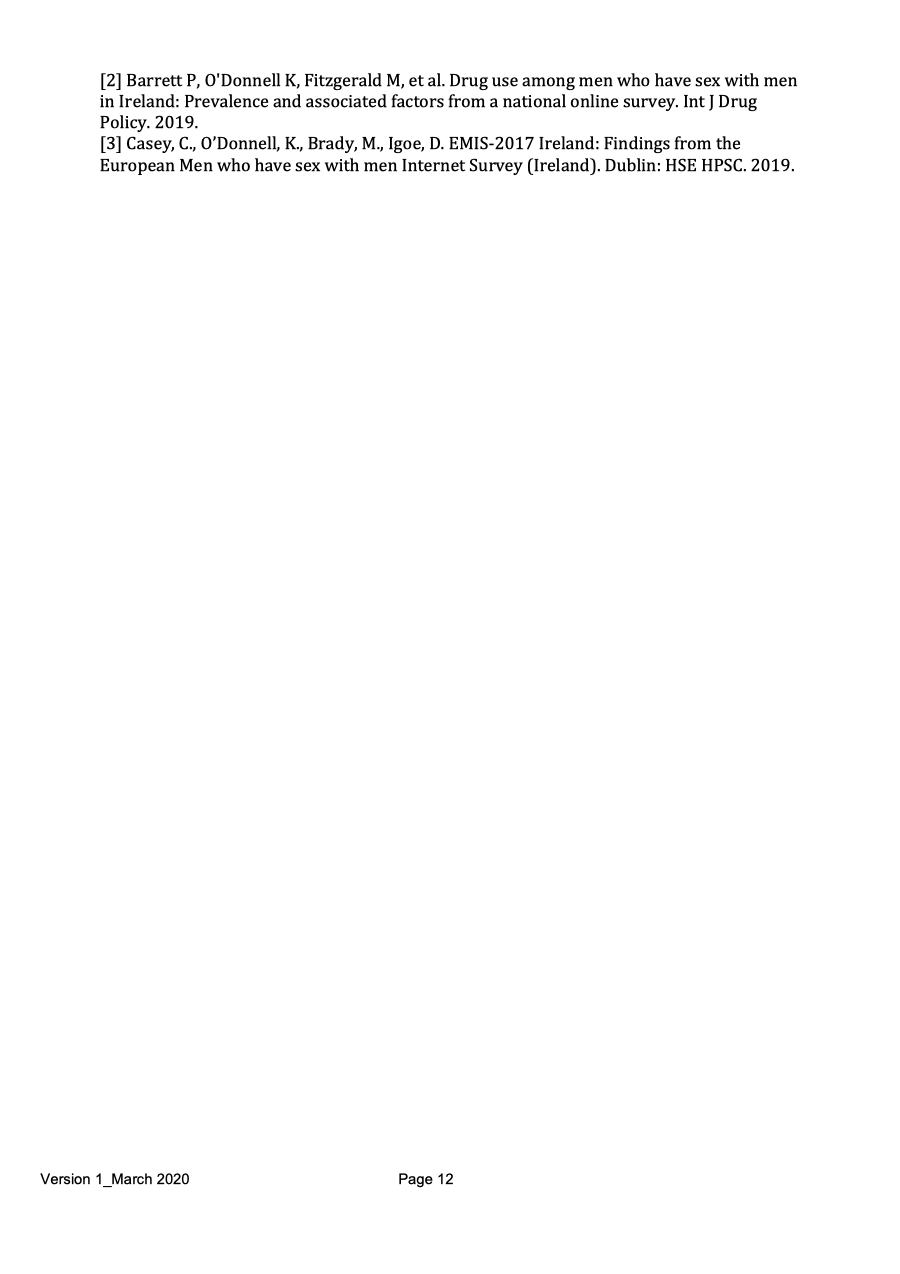

Supplement: S2 Appendix — (DOCX) [file pone.0288171.s002.docx]
