# Supplementary material for: Recreational and sexualised drug use among gay, bisexual, and other men who have sex with men (gbMSM) in Ireland–Findings from the European MSM internet survey (EMIS) 2017
Source: PLoS One. 2023 Jul 28;18(7):e0288171. doi: 10.1371/journal.pone.0288171 (PMC10381075; doi:10.1371/journal.pone.0288171)
Supplement: S3 Appendix — (DOCX) [file pone.0288171.s003.docx]

# Appendix C – Prevalence of past-year use of each specific drug included in the “recreational drug use” variable, by HIV-status:

|  | **No HIV diagnosis**  **n (%)** | **Diagnosis of HIV**  **n (%)** |
| --- | --- | --- |
| Cannabis | 639/1898 (33.7) | 54/141 (38.3) |
| Cocaine | 379/1895 (20.0) | 38/141 (27.0) |
| MDMA pill form | 342/1898 (18.0) | 44/141 (31.2) |
| MDMA powder or crystal form | 284/1895 (15.0) | 25/140 (17.9) |
| Ketamine | 155/1897 (8.2) | 18/141 (12.8) |
| Amphetamine/Speed | 129/1895 (6.8) | 14/141 (9.9) |
| GHB/GBL | 125/1896 (6.6) | 27/141 (19.1) |
| LSD | 68/1895 (3.6) | 5/141 (3.5) |
| Crystal methamphetamine | 57/1894 (3.0) | 22/141 (15.6) |
| Synthetic cannabinoids | 29/1893 (1.5) | 3/141 (2.1) |
| Mephedrone | 25/1895 (1.3) | 5/141 (3.5) |
| Synthetic stimulants other than mephedrone | 18/1897 (0.9) | 3/141 (2.1) |
| Crack cocaine | 11/1897 (0.6) | 0/141 (0.0) |
| Heroin or other related drugs | 9/1898 (0.5) | 2/141 (1.4) |
